# Exergame (ExerG)-Based Physical-Cognitive Training for Rehabilitation in Adults With Motor and Balance Impairments: Usability Study

**DOI:** 10.2196/66515

**Published:** 2025-02-14

**Authors:** Silvia Herren, Barbara Seebacher, Sarah Mildner, Yanick Riederer, Ulrike Pachmann, Nija Sonja Böckler, Stephan Niedecken, Sabrina Alicia Sgandurra, Leo Bonati, Isabella Hotz, Alexandra Schättin, Roman Jurt, Christian Brenneis, Katharina Lenfert, Frank Behrendt, Stefan Schmidlin, Lennart Nacke, Corina Schuster-Amft, Anna Lisa Martin-Niedecken

**Affiliations:** 1 Research Department, Reha Rheinfelden, Rheinfelden, Switzerland; 2 Department of Rehabilitation Science, Clinic for Rehabilitation Muenster, Gröben 700, Muenster, 6232, Austria, 43 533720004 ext 6205; 3 Clinical Department of Neurology, Medical University of Innsbruck, Innsbruck, Austria; 4 Karl Landsteiner Institute for Interdisciplinary Rehabilitation Research, Muenster, Austria; 5 Sphery Ltd, Zurich, Switzerland; 6 VASCage – Centre on Clinical Stroke Research, Innsbruck, Austria; 7 Department of Design, Institute for Design Research, Zurich University of the Arts, Zurich, Switzerland; 8 English Language and Literature, Faculty of Arts, University of Waterloo, Waterloo, ON, Canada; 9 Stroke Center and Department of Neurology, University Hospital Basel, Basel, Switzerland; 10 Department of Clinical Research, University of Basel, Basel, Switzerland; 11 Department of Neurology, Clinic for Rehabilitation Muenster, Muenster, Austria; 12 School of Engineering and Computer Science, Bern University of Applied Sciences, Burgdorf, Switzerland; 13 HCI Games Group, Stratford School of Interaction Design and Business, University of Waterloo, Waterloo, ON, Canada; 14 Department for Sport, Exercise and Health, University of Basel, Basel, Switzerland

**Keywords:** exergame, rehabilitation, user-centered design, usability testing, mixed-methods, interdisciplinary research, concept functional model proofs, exercise, cognitive training, technology acceptance, motor, cognitive impairment, safety, user experience, balance impairments, balance

## Abstract

**Background:**

Exergames are increasingly used in rehabilitation, yet their usability and user experience for patients and therapists, particularly for functional model systems, are underresearched. The diverse needs and preferences of users make conducting usability studies challenging, emphasizing the need for further investigation in real-world settings.

**Objective:**

This study aimed to evaluate the usability, safety, and user experience of a novel exergame functional model, the ExerG, from the perspectives of patients and therapists in a rehabilitation setting.

**Methods:**

In this mixed methods study, 15 patients undergoing rehabilitation (primary end users [PEUs]) and 20 therapists (secondary end users [SEUs]) from 2 rehabilitation centers in Switzerland and Austria participated in exercising and observation sessions with the ExerG. SEUs received training on system use and technical issue management, enabling them to fulfill their therapist roles while treating patients or mock patients. Rapid Iterative Testing and Evaluation was used and the training software adjusted based on participant feedback. Usability was assessed with questionnaires, semistructured interviews, and through observations during the ExerG testing. System acceptability was evaluated using specific quantitative thresholds based on PEU performance and feedback. An observation protocol tracked SEUs’ correct use, errors, hesitations, task completion time, and needed assistance across scenarios.

**Results:**

Patients and therapists reported overall good usability and positive experiences with the exergame. PEUs rated 23/29 (79%) instructions as acceptable, showed good-to-very-good exercise performance in 19/29 (65%) tasks, and completed 28/29 (97%) tasks. Patients reported no adverse events, showing improved performance and enjoyment across ExerG exercising rounds, with 79/90 (88%) expressing positive emotions and reporting median scores of 9 (IQR 7.5‐10) on a 1‐10 user satisfaction scale. Patients were willing to continue using the device if the graphic design was improved (5/15), tracking systems and projector quality were enhanced (each 3/15), instructions clarified (12/15), and the game variety increased (2/15). PEUs felt secure in the safety harness (15/15) but recommended swivel arm movement enhancements (5/15). SEUs effectively executed scenarios, with hesitation and difficulties observed in only 14/41 tasks and 2/41 tasks, across all 20 therapists, accounting for 1.7% and 0.2% of the 820 total task cases, respectively. Therapists’ quantitative usability ratings were high (median System Usability Scale score 82.5, IQR 65‐95). All SEUs expressed their willingness to use the ExerG (20/20) and reported being able to operate the system using the user handbook (20/20). They emphasized the motivation-enhancing effect of video-game based training (12/20) and considered the activities supportive for physical and cognitive skills (20/20). They suggested incorporating daily living task simulations (13/20), more customizable options (6/20), more targeted motivational feedback (9/20), clearer performance ratings (9/20), and more concise activity instructions (6/20).

**Conclusions:**

The interdisciplinary, iterative ExerG development approach shows promise. The findings will inform future optimizations. Future work will assess long-term impact.

## Introduction

The rapid advancement of digital technologies has revolutionized various aspects of health care, including rehabilitation. Among these innovations, exergames—video games that require physical activity to play—have emerged as promising tools for engaging patients in rehabilitative exercises. Exergames use physical activity and brain challenges to boost patient motivation, adherence, and results in rehabilitation settings [1,2].

Despite the growing interest in exergames for rehabilitation, there is a lack of research on their usability and user experience, particularly for functional model systems [3,4]. A functional model is a representation that describes how a system, device, product, or process operates in terms of its functions and the interactions between its components [5]. It focuses on the purposes of different parts and how they contribute to the overall objectives of the system [5]. Usability, which refers to the effectiveness, efficiency, and satisfaction with which users can achieve specific goals in a particular environment [6,7], is a critical factor in the adoption and success of rehabilitation technologies. Poor usability can lead to frustration, reduced motivation, and even abandonment of the technology [8]. Similarly, user experience, which encompasses the user’s perceptions, emotions, and responses to the technology [9], can significantly influence patient engagement and adherence to rehabilitative interventions [8,10].

Evaluating the usability and user experience of exergame functional models is essential for iterative design and optimization. Functional model testing allows developers to identify and address usability issues early in the development process, reducing the risk of costly redesigns and improving the final product’s quality [11]. However, conducting usability studies with rehabilitation populations presents challenges due to the diverse needs, abilities, and preferences of both patients and therapists [12]. Key considerations include safety, training goals, individuality, game environment, social interactions, and physical and technical overload for patients, as well as facets like meaningfulness, distractions from the game environment, safety, gamification elements, and the availability and accessibility of the exergame for therapists [13]. Therefore, further investigation into the usability and user experience of exergame functional models in real-world rehabilitation settings is essential.

To address this gap, we developed a novel exergame functional model called ExerG, designed for use in rehabilitation. The ExerG incorporates a range of physical-cognitive training elements, including balance, coordination, and dual-tasking exercises, which are crucial for rehabilitation [14]. The development of the ExerG followed an interdisciplinary, iterative approach, involving collaboration between game designers, rehabilitation experts, and primary end users (PEUs; patients) and secondary end users (SEUs; therapists) [15]. This approach allows for the integration of clinical knowledge, user feedback, and technical expertise to create a user-centered design that meets the specific needs of the rehabilitation context [16,17].

The primary aim of this study was to evaluate the usability, safety, performance, and user experience of the ExerG functional model in a rehabilitation setting. We hypothesized that ExerG would demonstrate good overall usability and positive user experiences for both patients and therapists, despite the challenges associated with its functional model status. Additionally, we aimed to identify areas for optimization and inform future iterations of the ExerG system [12,18].

## Methods

### Study Design

To achieve this study’s objectives, we conducted a mixed methods study involving patients (PEUs) and therapists (SEUs) from 2 rehabilitation centers in Switzerland and Austria. This summative usability study employed a convergent mixed methods design to comprehensively evaluate the usability, safety, performance, and user experience of ExerG, a novel training solution for rehabilitation in adults with motor and balance impairments [12,18]. Summative usability testing is conducted to assess whether intended users can use the device safely and effectively in its intended environments [19]. The Good Reporting of a Mixed Methods Study (GRAMMS) framework [20] was adhered to (Multimedia Appendix 1). Our testing procedures adhered to the International Organization for Standardization’s guidelines for usability engineering of medical devices (IEC 62366-1:2015 and IEC TR 62366‐2). We used the Rapid Iterative Testing and Evaluation Method [21], which enabled swift adjustments to the training software based on participant feedback. This approach enabled continuous refinement of the system throughout this study, ensuring responsiveness to user needs and enhancing the ExerG’s overall usability.

### Exercise and Test Items

The new training solution comprises the ExerG training software for patients, the ExerCube supporting material, and the safety harness for the ExerG. The ExerG training solution is intended to be operated by professionals (therapists/SEUs) and used by patients (PEUs). The ExerG exercise program integrates a variety of physical-cognitive training elements, including balance, coordination, and dual-tasking, through engaging mini-games such as apple picking, pattern matching, rowing, trunk jumping, and balloon catching. These activities are designed to enhance coordinative skills by refining precise spatial and temporal movements of the legs and arms, as well as improving the control and regulation of motor activity in the central nervous system. They also promote conditional abilities by requiring dynamic balance during fast leg movements to help prevent falls. Therapists can select exercises, personalize settings before each session, and make real-time adjustments to tailor the program to each individual’s needs. Training intensities vary from easy to hard.

The exercise program features a range of activities that progress in difficulty. Walking variations include low to high leg lifts at different speeds and dual-task walking with complex arm movements. Side steps incorporate arm movements and responses to stimuli, while front steps and lunges vary from small to large movements, and squat depths increase. Jumping exercises advance to include jumps that break floor contact, and the swaying tree pose becomes more challenging. Balance exercises include single-leg stances and full leg lifts, with symmetrical and asymmetrical arm tasks, along with body rotations. Swimming arm movements speed up, and challenges involve reaching for wall stimuli. Balloon-catching activities vary in speed, while obstacle jumps progress from galloping to standard jumps, and fruit sorting speeds change from slow to fast (Multimedia Appendix 2).

**Figure 1.**
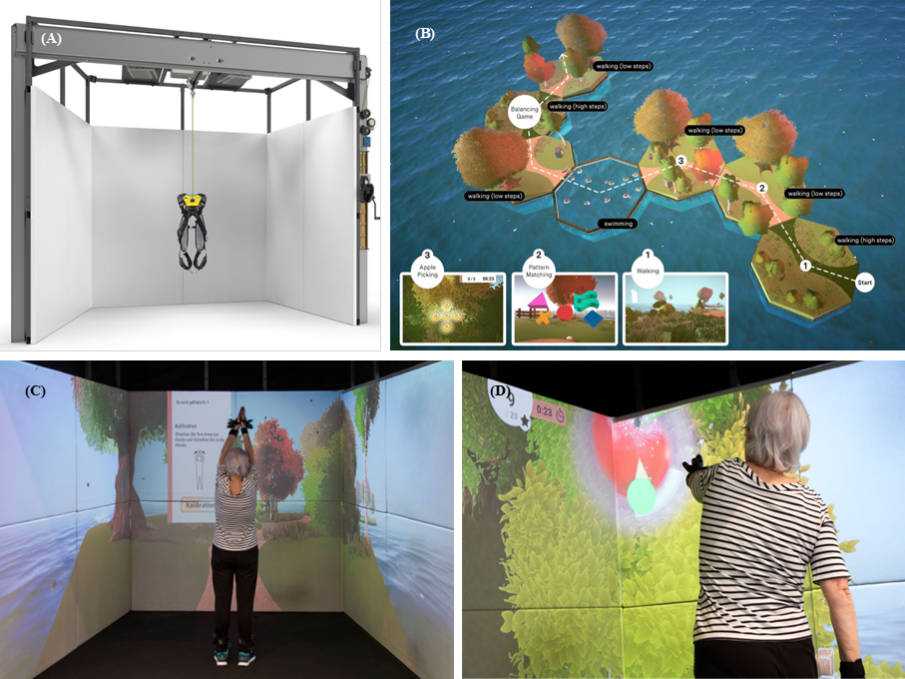
(A) Overview of the ExerG hardware, (B) scenarios overview, (C) trackers calibration of the system for the user, and (D) the apple picking scenario.

The ExerG training software and the safety harness are complementary and adapted technologies of the ExerCube, an already existing immersive fitness training device [22] (Multimedia Appendix 2). In combination with the safety harness (Petzl; Figure 1A), its purpose is to enable training in a safe environment and mitigate the risk of patients falling (Figure 1B-D).

### Patients and Participants

#### Participant Recruitment

Recruitment took place from May 16, 2023, to October 31, 2023. Using convenience sampling, our study involved 2 user groups: patients (PEUs) and therapists (SEUs, participants), from rehabilitation centers in Switzerland (Reha Rheinfelden) and Austria (Clinic for Rehabilitation Muenster).

#### Primary End Users

Patients from inpatient, outpatient, and Neuro Day Care units were considered at 1 of the 2 centers. Recruitment involved screening entry lists, reviewing treated patients, distributing flyers, emailing outpatient physiotherapists, and engaging therapists. Medical records were screened for basic eligibility, and eligible patients received verbal and written study information. They had at least 24 hours to decide and sign the consent form if they were interested. See Textbox 1 for eligibility criteria. For more details on the Mini Mental State Examination (MMSE) and Berg Balance Scale, see Multimedia Appendix 3.

Textbox 1. **Eligibility criteria of primary end users.**

**Inclusion criteria**

- Aged ≥18 years
- Undergoing inpatient or outpatient rehabilitation
- Motor impairment of the upper or lower extremities, gait disorder, balance disorder, visual-spatial disorder, or cognitive disorder due to any disease
- Ability to speak and comprehend German and understand digitally transmitted training instructions
- No prior training experience with the device
- Body height ranging from 160 cm to 200 cm, in accordance with hardware specifications
- Body weight not exceeding 120 kg, as per the safety harness specifications
- Capable of performing the movements required for video game-based training. This includes independently transitioning from a sitting to a standing position (scoring ≥3 on the Berg Balance Scale [BBS] [23] item 1, “sitting to standing”) and maintaining the standing position without assistance (scoring ≥3 on the BBS item 2, “standing unsupported”)
- Capable of participating in the entire clinical investigation, as determined by this study’s principal investigator

**Exclusion criteria**

- Moderate-to-severe cognitive impairment as defined by a Mini Mental State Examination (MMSE) [24] score of ≤18 [25]
- Known cybersickness
- Severe visual, neurological, cardiorespiratory, psychiatric, or orthopedic impairments that reduce a person’s ability to follow instructions or play the games
- Epileptic seizures within the past 3 months
- Recent surgery, fractures, joint replacement, or malignancy within the past 3 months
- Impairment of hearing resulting in the inability to engage in verbal communication
- Severe movement pain exceeding 5 on the 11-point pain Numeric Rating Scale (NRS) [26]
- Physical conditions that prevent the proper wearing of the safety harness or could result in pain or health complications, such as skin lesions or open wounds (eg, severe osteoporosis)
- Joint contractures (eg, in the shoulder, knee, or hip) that may result in limitations during end user evaluation of the ExerG
- Severe neurological conditions (eg, severe epilepsy, advanced Parkinson disease, or condition after severe stroke)
- Severe psychiatric conditions (eg, pronounced paranoid states or severe depression)
- Terminal illness (estimated life expectancy <12 months)

#### Secondary End Users

SEU recruitment included physiotherapists, occupational therapists, training therapists, and sports scientists from the rehabilitation centers and external clinics. This diverse sampling approach ensured a comprehensive evaluation of the ExerG across various settings and professional perspectives. SEUs were required to be at least aged 21 years and hold a therapist qualification or a relevant bachelor’s degree.

#### Sample Size

This usability study sample included 15 PEUs and 20 SEUs. The SEU group emphasized diverse recruitment, engaging staff from various therapeutic disciplines with distinct specializations. Further, 5 therapists tested the ExerG in real-world scenarios with inpatients and outpatients. To balance sample sizes, an additional 10 PEUs were enrolled. Saturation was evaluated using code meaning, which entailed reviewing each interview to document identified codes and checking subsequent interviews for any new aspects, dimensions, or nuances of those codes until saturation was reached with no new information [27].

### Study Procedures

#### Overview

PEUs participated in an initial eligibility evaluation and baseline data collection, followed by a single session with 2 exercising rounds to test the ExerG and safety harness. SEUs, who were already familiar with technology due to their daily work, received training on system usage and technical issue management. This training included detailed explanations of their responsibilities while treating patients or mock patients during the evaluation session, with real individuals acting as stand-ins for actual patients (see the figure in the Results section for a flow chart).

#### Evaluation Session for Primary End Users

PEUs attended a single study visit with 2 training rounds. At the start of each training round, participants received an overview of the training content and procedures. Researchers collected demographic and disease-specific data and conducted baseline assessments. Patients were then briefed on the gaming activities and fitted with safety harnesses and wrist and ankle motion sensors. Once secured, the first training round began, lasting approximately 10‐15 minutes, depending on the patient’s physical and cognitive capabilities.

#### Evaluation Session for Secondary End Users

SEUs from both centers participated in a study visit that included ExerG usage training and 2 observation rounds. Before the training, SEUs received the system manual and were advised to read it thoroughly. During the session, they attended an introduction and training on system usage, following a standardized protocol for explanation and demonstration, and practiced procedures for memorization.

In the usage observation rounds 1 and 2, SEUs set up the system, designed a physical-cognitive training session using the software’s exergames, prepared a patient with training materials, and guided them through the session. This study’s team observed and documented the process. At the conclusion, SEUs completed rating scales and questionnaires, with the key difference being that 1 center used mock patients during the observation rounds.

### Data Collection

#### Overview

Data were collected during and after the PEU and SEU study visits using an observation protocol, questionnaires, and interviews. Quantitative data on usability, user experience, and acceptance were gathered with standardized rating scales. Qualitative measures, including semistructured interviews and specific questions about ExerG training scenarios, provided a more comprehensive assessment.

#### Primary Outcomes

This usability study focused on system interaction and safety, encompassing both software and hardware as the primary outcome. Adverse events were monitored throughout this study. To identify difficulties, problems, or errors during use, the team followed a detailed observation protocol with predefined categories. Both end user groups provided objective measurements (scale ratings) and subjective feedback as part of the protocol. Performance was assessed by calculating the proportion of participants who completed or met the criteria for each exercise. The test moderator evaluated exercise performance using an observation sheet with the following ratings: very good, good, half-half, poor, and very poor. Performance was categorized as “very good” if the sum of “very good” and “good” ratings was ≥85%. Tasks were aligned with the training program flow, assessing instruction clarity, exercise performance, task completion, and exercise intensity. The system’s acceptability was evaluated using specific quantitative thresholds based on PEU performance and feedback. The system was deemed acceptable by meeting the following criteria: (1) training completion: at least 85% of PEUs successfully completed the first training round; (2) exercise skipping: fewer than 20% of exercises were skipped by PEUs; (3) instruction comprehensibility: at least 85% of PEUs rated their understanding of the training instructions as “well” or “very well”; and (4) performance: at least 85% of PEUs performed the physical and cognitive activities with “good” to “very good” performance ratings.

For SEUs, an observation protocol recorded correct use, errors, hesitations, task completion time, and needed assistance across 8 scenarios: hardware preparation, safety system preparation or attachment, fall simulation, initiating or supporting training, compiling training, ending training, removing safety system, and shutting down hardware. “Hesitation” implied uncertainty or a lack of confidence, while “difficulties” represented objective barriers (MultimediaAppendices 4  5).

#### Secondary Outcomes for Primary End Users

Following each training round, PEUs evaluated perceived physical exertion and mental effort using the Borg CR10 (Borg Category Ratio Scale) [28] and the Paas Mental Effort Rating Scale [29]. They assessed player experience using the Player Experience Inventory (PXI) [30,31], focusing on both functional and psychosocial aspects of the exergame (Multimedia Appendix 3). Semistructured interviews and observations were also conducted for deeper insights into exergame functionality.

At the end of their study visit, PEUs participated in a brief semistructured interview featuring both structured and open-ended questions to gather insights into their perceptions of game control and comfort. Throughout the exergaming training, this study’s team used an observation sheet with predetermined categories for flow, motivation, and motion sickness, documenting all training activities in sequence. This sheet included checkboxes for ratings on:

- Level (easy, medium, or hard)
- Execution (yes, no, or skipped)
- Clarity of instructions (very good, good, half-half, poor, or very poor)
- Ability to execute the required movements (very good, good, half-half, poor, or very poor)
- Training intensity (too difficult, difficult, optimal, easy, or too easy)

Additionally, a study team member evaluated the perceived helpfulness of the exergame feedback using a rating scale (very good to very poor). A trained member also systematically observed and noted the patient’s emotions during gameplay [32]. Any emotions and difficulties observed or reported by the patient during or after the training were recorded and discussed to analyze their causes and potential solutions [32] (Multimedia Appendix 3).

#### Secondary Outcomes for Secondary End Users

SEUs evaluated the ExerG using the System Usability Scale (SUS) [33], a common questionnaire for assessing technology usability. The SUS includes 10 items with alternating positive and negative statements, each rated on a 5-point Likert scale, and is widely used in human-computer interaction research. According to Bangor et al [34], SUS scores of 70‐79 indicate good or acceptable usability, while scores of 80‐89 indicate excellent usability.

After completing all use scenarios, any difficulties encountered were discussed with SEUs. A semistructured interview, featuring both structured and open-ended questions, was conducted after the second ExerG usage observation round to evaluate user-friendliness and comprehensively assess SEUs’ experience with the device, focusing on exercise content, relevance to everyday life, and usability.

### Data Analyses

#### Statistical Data Analysis

Demographic and usability metrics were analyzed using descriptive statistics. Absolute and relative frequencies were calculated for count and nominal data. Ordinal data (MMSE, Borg CR10, Paas Mental Effort Rating Scale, and SUS) are presented with medians and IQRs, which reflect the range between the 25th and 75th percentiles of the data. Continuous data are reported with means and SDs. The usability problem discovery rate was calculated by dividing the number of identified problems by the total number of possible problems and then multiplying by 100 to obtain a percentage. Statistical analysis was performed using IBM SPSS software (version 28.0; IBM Corp) and GraphPad Prism 9 (GraphPad Software).

#### Qualitative Data Analysis

The patients’ responses from the brief interview were recorded and subsequently entered into an Excel (Microsoft Corp) spreadsheet for further analysis. Aligning with the principles of qualitative content analysis as outlined by Mayring [35,36], and following familiarization with the material, a coding scheme was developed inductively, based on the data and the research question. This process resulted in categories that were unidimensional, mutually exclusive, and exhaustive. Clear coding rules were established to represent recurring themes, ideas, and concepts within the texts. Each condensed phrase was assigned to one or more codes from the coding scheme, ensuring a thorough classification of segments. Through an iterative process, the codes and their assignments were reviewed and discussed by 2 researchers to enhance intersubjective conformability and ensure reliability [37]. The codes were applied to relevant segments of text, ensuring that each segment was accurately classified according to the established categories [37]. The frequencies of the codes were tallied, and the coded data were synthesized to create overarching themes that captured the key insights from the analysis. Themes were then described and illustrated with quotations accompanied by corresponding participant IDs.

### Ethical Considerations

This study was approved by the Research Ethics Committee of Northwestern and Central Switzerland (IRB approval: amendment3_2022‐00559), and informed consent was obtained from participants, who were able to opt out at any time. Participants’ data were anonymized. In Austria, this study did not require Ethics Committee approval as it involved only therapists and focused on hardware and software handling. No compensation was provided to participants in this research study. This study was prospectively registered (ClinicalTrials.gov ID NCT05967078; OSF Preregistration OSF.IO/CQ9AT).

## Results

### Patients’ and Participants’ Characteristics

**Figure 2.**
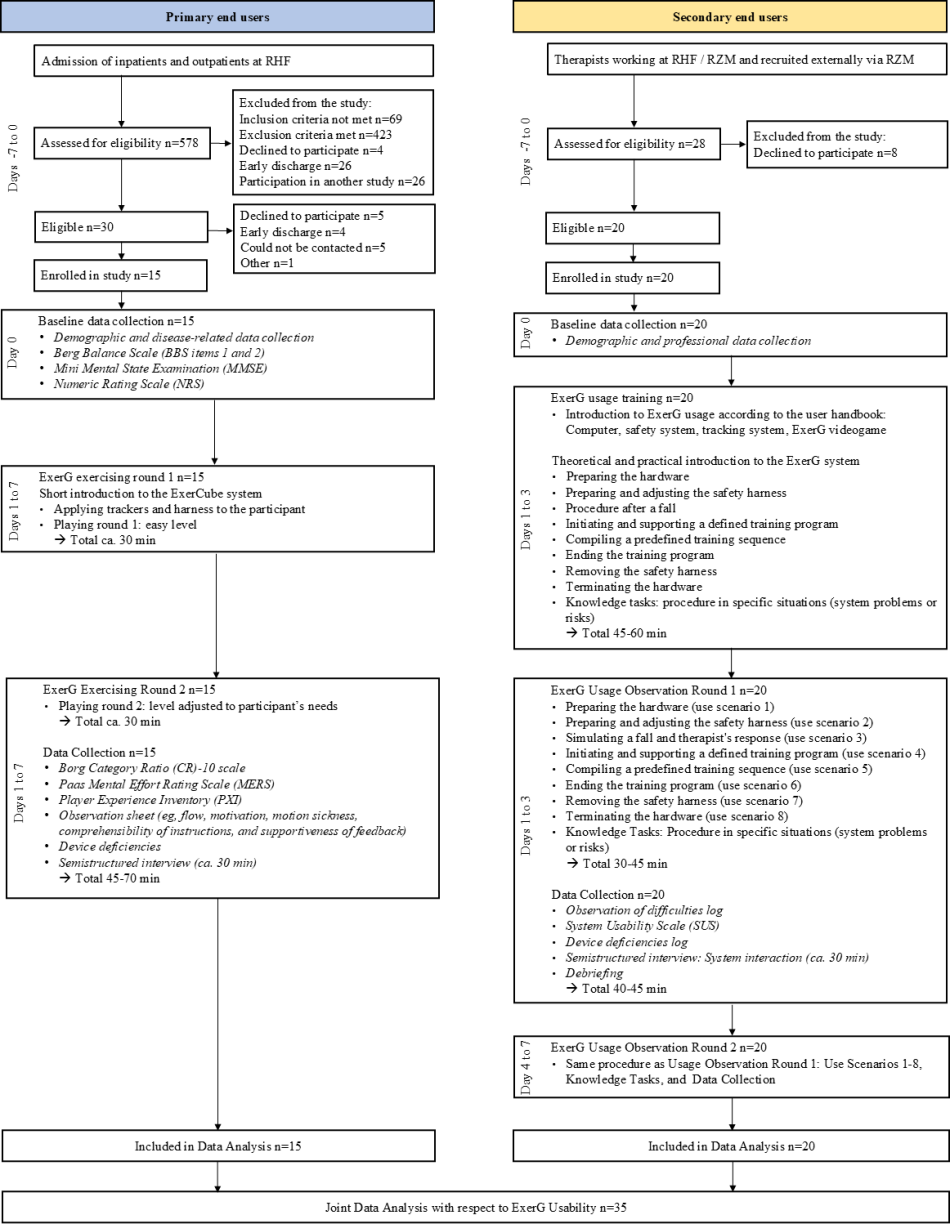
Study flow diagram. RHF: Reha Rheinfelden; RZM: Reha Zentrum Muenster.

A total of 15 PEUs and 20 SEUs were recruited across both study centers (Figure 2).

#### Primary End User Group

In total, 15 patients, with neurologist-assessed motor and balance impairment, exhibiting a mean age of 57.8 (SD 12.25) years, completed this study (Table 1). This study’s population comprised 3 patients using walking aids and 5 with a history of falls. Among the PEUs, 11 (73%) had prior experience with technology-based training, primarily familiar with exergames and stationary weight-relieving systems. Only 1 (7%) PEU was acquainted with both mobile body-weight-supporting systems and virtual reality.

**Table 1.** Primary end user characteristics.

	Values (n=15)
Age (years), mean (SD; range)	57.8 (12.25; 40‐78)
Sex, n (%)	
Male	9 (60)
Female	6 (40)
Mobility aids, n (%)	
None	12 (80)
Walking sticks	1 (6.7)
Walker	2 (13.3)
Fallers, n (%)	5 (33.3)
Nonfallers, n (%)	10 (66.7)
Falls in the previous 5 months, n (%)	
None	10 (66.7)
1 time	4 (26.7)
1‐5 times	1 (6.7)
MMSE^a^ score, median (IQR; range)	29 (28‐30; 22‐30)
Primary diagnoses, n (%)	
Multiple sclerosis	4 (26.7)
Lacunar thalamo-capsular stroke	2 (13.3)
Middle cerebral artery stroke	1 (6.7)
Brainstem stroke	1 (6.7)
Cerebellar stroke	1 (6.7)
Meningoencephalitis	1 (6.7)
Orthostatic tremor	1 (6.7)
Pneumococcal sepsis	1 (6.7)
Progressive gait disorder	1 (6.7)
Dermatomyositis	1 (6.7)
Guillain-Barré syndrome	1 (6.7)
Secondary diagnoses (multiple), n (%)	
Arterial hypertonia	3 (20)
Atherosclerosis	1 (6.7)
Chronic renal insufficiency	1 (6.7)
Diabetes mellitus	1 (6.7)
Dyslipidemia	1 (6.7)
Dyslipoproteinemia	1 (6.7)
Episodic migraine	1 (6.7)
Meningoencephalitis	1 (6.7)
Mild obesity	1 (6.7)
Multiple sclerosis	1 (6.7)
Progressive gait ataxia	1 (6.7)
Recurrent depressive mood disorder	1 (6.7)
Sensorimotor polyneuropathy	1 (6.7)

a MMSE: Mini Mental State Examination.

#### Secondary End User Group

A total of 20 therapists and sports scientists participated in and completed this study (Table 2).

**Table 2.** Secondary end user characteristics.

	Values (n=20)
Age (years), mean (SD; range)	33.61 (9.4; 23.7‐59.3)
Sex, n (%)	
Male	6 (30)
Female	14 (70)
Professional experience (years), mean (SD; range)	8.9 (8.9; 1‐38)
Profession, n (%)	
Physiotherapist	14 (70)
Occupational therapist	3 (15)
Sport scientist	3 (15)
Professional qualification, n (%)	
Vocational diploma	4 (20)
Bachelor’s degree	7 (35)
Master’s degree	7 (35)
Doctoral degree	2 (10)
Professional field (multiple), n (%)	
Neurological rehabilitation	16 (80)
Orthopedic rehabilitation	3 (15)
Geriatric rehabilitation	5 (25)
Medical fitness	2 (10)
Internal medicine, oncological rehabilitation	4 (20)
Psychomotor rehabilitation	3 (15)
Mixed	3 (15)

### Primary Outcomes

#### Primary End User Group

No adverse events were reported during this study. Table 3 summarizes the activities per exercising round, detailing the clarity of instructions, PEUs’ exercise performance, and completion rates, including activity intensity for ExerG exercising round 2. PEUs became familiar with the tasks in round 1, leading to improved performance in round 2. However, newly introduced activities continued to pose challenges, with some usability issues noted. Overall, 23 of 29 (79%) instructions were rated acceptable, good-to-very-good exercise performance was observed in 19 of 29 (65%) tasks, and 28 of 29 (97%) tasks were completed.

**Table 3.** Primary end user—ExerG software interaction.

Session and task	Clarity of instructions	Exercise performance	Task completion	Exercise intensity	
	Good to very good	Good to very good	Yes	Optimal	(Very) easy
ExerG exercise round 1					
System calibration	93.3^a^	93.3^a^	93.3^a^	—^b^	—
Walking	73.3	80	100^a^	—	—
Apple picking	93.3^a^	86.7^a^	93.3^a^	—	—
Walking	86.7^a^	80	100^a^	—	—
Pattern matching	86.7^a^	86.7^a^	100^a^	—	—
Walking	93.3	86.7^a^	100^a^	—	—
Rowing	73.3	60	80	—	—
Tall grass walking	66.7	73.3	100^a^	—	—
Apple picking	100^a^	60	100^a^	—	—
Walking	100^a^	100^a^	100^a^	—	—
Pattern matching	100^a^	93.3^a^	100^a^	—	—
Walking	100^a^	100^a^	100^a^	—	—
ExerG exercise round 2					
System calibration	93.3^a^	93.3^a^	93.3^a^	0	93.3^a^
Walking	100^a^	93.3^a^	100^a^	20	80
Apple picking	93.3^a^	80	100^a^	13.3	86.7^a^
Walking	100^a^	100^a^	100^a^	20	80
Trunk jumping	80	86.7^a^	93.3^a^	40	33.3
Walking	100^a^	100^a^	100^a^	13.3	86.7^a^
Rowing	100^a^	93.3^a^	100^a^	20	80
Walking	100^a^	100^a^	100^a^	13.3	80
Pick apples, avoid nuts	80	60	100^a^	40	26.7
Walking	100^a^	100^a^	100^a^	13.3	86.7^a^
Tall grass walking	93.3^a^	86.7^a^	100^a^	26.7	53.3
Walking	100^a^	100^a^	100^a^	13.3	86.7^a^
Time-limited pattern matching	100^a^	80	100^a^	33.3	66.7
Walking	100^a^	100^a^	100^a^	13.3	86.7^a^
Catching balloons	60	80	93.3^a^	40	26.7
Walking	100^a^	100^a^	100^a^	13.3	86.7^a^
Multitasking in apple picking	93.3^a^	60	100^a^	40	46.7

a These numbers indicate values above the 85% acceptance criteria.

b Not applicable.

Figure S2 in Multimedia Appendix 4 illustrates the emotions observed during PEU exercise activities. Enjoyment, curiosity, attention, and amazement were categorized as positive emotions, accounting for 79 of 90 (88%) of the expressed emotions during training. Some PEUs exhibited negative emotions, such as nervousness, boredom, and confusion, but none expressed anger or resistance. Perceived physical and mental effort increased with higher exercise levels, with physical exertion generally viewed as greater than mental effort (Figure S3 in Multimedia Appendix 4).

#### Secondary End User Group

Analysis of the observation protocol revealed that most SEUs successfully executed the use scenarios. Minor difficulties or hesitation were noted in 14 of 41 tasks and difficulties in 2 of 41 tasks among the 20 therapists accounting for 1.7% and 0.2% of the total 820 task cases, respectively. Assistance from this study’s personnel was required in 9 instances across all scenarios (1.1%), with no use errors detected. Independent, correct, and effective use was observed for all tasks in fall simulation (3), supporting training (4), compiling training (5), removing the safety system (7), and shutting down hardware (8). The mean time to complete tasks was 2.6 (SD 3.8) minutes, indicating the clarity and effectiveness of the ExerG user handbook and ExerG usage training (Tables4  5).

**Table 4.** Secondary end user—ExerG hardware and software interaction across use scenarios 1‐4 (n=20).

Use scenarios and tasks for secondary end users	Use^a^	Support^a^
Preparing the hardware (use scenario 1)^b^		
Turn on main power switch	✓	No
Turn on computer	✓	No
Turn on touchscreen or check if it is on	Difficulties 1	Yes 1
Turn on the keypad or check if it is on	✓	No
Turn on the 3 projectors using the remote	Hesitation 1	No
Select correct HDMI channels for projectors with remote	Hesitation 2	Yes 1
Enter the computer password	✓	No
Check internet connection	✓	No
Preparing and attaching the safety system (use scenario 2)^c^		
Check proper rope position and ensure it is undamaged	Hesitation 2	No
Perform the end stop test	Hesitation 2	No
Properly don the Petzl Newton Fast harness (correct size)	✓	No
Correctly connect the Petzl harness system	Hesitation 1	No
Tighten the rope if necessary	✓	No
Adjust the drop height correctly using the crank handle	✓	No
Simulating a fall scenario (use scenario 3)^d^		
If the patient falls and is caught by the safety system:		
Move to chair to help them stand	✓	No
Lower to the floor to stand independently	✓	No
Initiating a predefined training program (use scenario 4A)^e^		
Remove the trackers from the charging station	✓	No
Attach trackers securely to person’s ankles and wrists	Hesitation. 1	Yes 1
Turn on the trackers (green light).	Hesitation 1	Yes 1
Select the ExerG training program from the menu	✓	No
Choose the predefined training sequence from the menu	Hesitation 2	Yes 2
Start the selected training program	✓	No
Properly guide the (mock) patient through calibration	✓	No
Supporting a predefined training program (use scenario 4B)^f^		
Accompany (mock) patient during exergaming training	✓	No

a With columns “use” and “support,” the numbers represent the number of therapists, who showed one of the following behaviors: independent, correct, and effective use indicated by a checkmark, and hesitation or experiencing difficulties.

b Mean total duration 1.6 (SD 1.8) minutes.

c Mean total duration 2.4 (SD 0.8) minutes.

d Mean total duration 0.9 (SD 0.5) minutes.

e Mean total duration 1.9 (SD 1) minutes.

f Mean total duration 12.7 (SD 2.7) minutes.

**Table 5.** Secondary end user—ExerG hardware and software interaction across use scenarios 5‐8 (n=20).

Use scenarios and tasks for secondary end users	Use^a^	Support^a^
Compilation of a predefined training sequence (use scenario 5)^b^		
Select the ExerG training program in the launcher	✓	No
Open a new training sequence	✓	No
Start editing mode	✓	No
Choose appropriate exercises	✓	No
Name the training sequence with the specified patient ID	✓	No
Save the training sequence	✓	No
Start the new training sequence	✓	No
Ending a training program (use scenario 6)^c^		
End the ExerG training program	Hesitation 2	Yes 1
Exit the user interface (launcher)	✓	No
Removing the safety system (use scenario 7)^d^		
Remove the wrist and ankle trackers	✓	No
Attach the 4 trackers to the charging station	✓	No
Crank down to release rope tension	✓	No
Release the rope from the Petzl harness system	✓	No
Loosen and remove the Petzl Newton Fast harness	✓	No
Terminating the hardware (use scenario 8)^e^		
Turn off the 3 projectors with the remote	Difficulties 1	Yes 2
Shut down the computer	✓	No
Switch off the main power switch of the system	✓	No

a With columns “use” and “support,” the numbers represent the number of therapists, who showed one of the following behaviors: independent, correct, and effective use indicated by a checkmark, and hesitation or experiencing difficulties.

b Mean total duration 1.9 (SD 0.6) minutes.

c Mean total duration 0.2 (SD 0.08) minutes.

d Mean total duration 1.5 (SD 0.6) minutes.

e Mean total duration 0.4 (SD 0.3) minutes.

SEUs demonstrated mastery of the knowledge tasks, reflecting clear user handbook instructions. These tasks included summarizing the product’s purpose, adjusting the video game volume, troubleshooting software issues (eg, user interface not opening or incidents during training), and managing ongoing activities (eg, skipping activities or restarting programs). Hardware tasks involved resolving issues with HTC Vive (HTC Corporation) cameras and trackers, ensuring proper recognition at startup, and correcting screen projections. Backup system tasks required knowledge of troubleshooting rope positioning and resistance during the end-stop test.

### Secondary Outcomes

#### Primary End User Group

**Figure 3.**
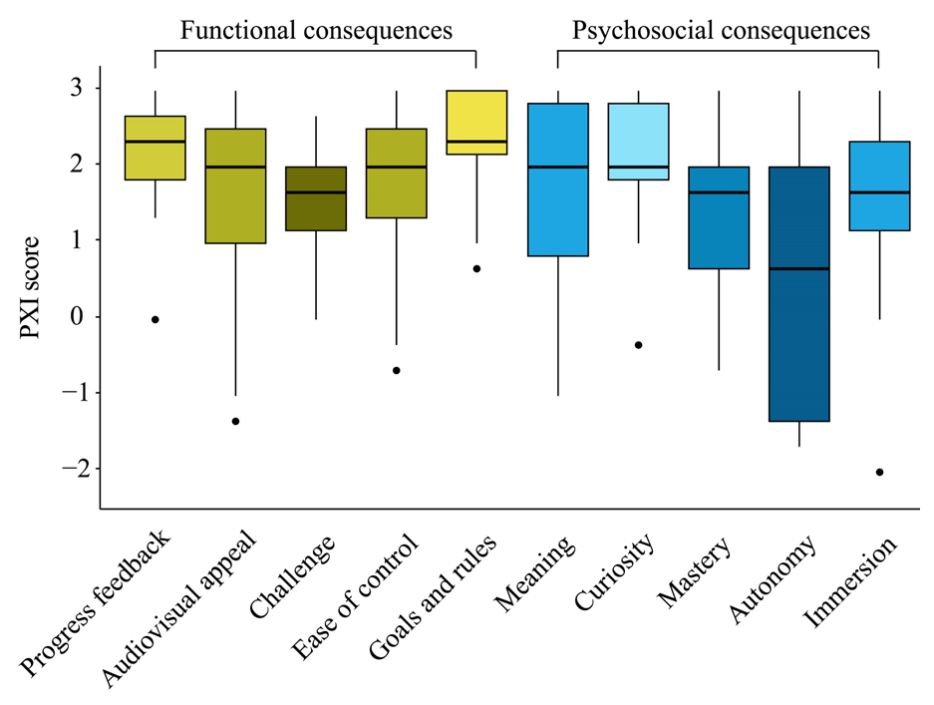
Ratings of patient player experience. PXI: Player Experience Inventory.

Ratings of patient player experience using the PXI indicated a positive or very positive experience. Functional consequences, such as audiovisual appeal or ease-of-control, were generally rated high. In contrast, related to psychosocial consequences, “autonomy” received the lowest score and showed a large IQR, suggesting that PEUs felt they lacked control over their engagement in the game. Conversely, perceived mastery was rated highly (Figure 3 and Multimedia Appendix 4), closely linked to the aspect of autonomy.

Figure 3 shows a boxplot of player experience across patients as assessed using the PXI, grouped by functional and psychosocial consequences. The boxes represent the IQR, with a median line, while whiskers extend to the minimum and maximum within 1.5 times the IQR, and outliers are indicated as points beyond this range.

Figure S4 in Multimedia Appendix 4 presents findings from the structured portion of the semistructured interview with PEUs. Most patients expressed enjoyment of the ExerG training, with positive feedback on the software and hardware. For the first question, PEUs rated their overall satisfaction at a median of 9 (IQR 7.5‐10) on a 1‐10 scale.

Saturation was achieved after 15 patient interviews, which revealed three themes through content analysis of the qualitative data: (1) enjoyment and acceptance, (2) live interaction, and (3) safety and comfort. Most patients enjoyed the training and would continue using the device (each n=12, 80%) if certain improvements were made (n=13, 87%), such as better graphic design (n=5, 33%), upgraded tracking systems (n=3, 20%), refined swivel arms (n=8, 53%), and enhanced projector quality (n=3, 20%). More variety in game activities and difficulty levels would also enrich the experience (n=2, 13%). While most users could understand the activity instructions (n=8, 53%) and feedback texts (n=9, 60%), they preferred shorter, more concise instructions with clear task-related wording and adequate reading time (n=12, 80%). Patients felt safe (n=15, 100%) and comfortable using the safety harness (n=13, 87%) but recommended improving the safety system, particularly the swivel arm’s movements, for smoother balance control (n=5, 33%). Many also noted limitations with exploring the game environment (n=11, 73%) and discomfort with the rope pulley during specific activities (n=3, 20%). Table S1 in Multimedia Appendix 6 presents theme descriptions and quotes.

#### Secondary End User Group

In SEUs, a median SUS score of 82.5 (IQR 18.7) was reported, indicating excellent usability of the ExerG [34]. The structured interview analysis showed that therapists were satisfied with the system (median score of 8, IQR 7‐8 on a scale of 1‐10), were willing to use it (n=20, 100%), and found it highly user-friendly (n=20, 100%). Less than half of the therapists found the feedback helpful (n=8, 40%), and one-fifth considered the video feedback to be comprehensive (n=4, 20%). SEUs indicated that the training content does not or only partly resembles everyday tasks (Figure S5 in Multimedia Appendix 4).

Saturation was achieved after 19 therapist interviews—content analysis of the qualitative data identified five themes: (1) acceptance and motivation, (2) meaningful activities, (3) training feedback, (4) individualization, and (5) safety and autonomy. Therapists noted that video-game based training could boost patient motivation through its engaging design and surprising elements, making it more enjoyable than standard therapy (n=12, 60%). They found the activities beneficial for physical and cognitive functions (n=20, 100%) but recommended incorporating more exercises that simulate daily living tasks for improved relevance (n=13, 65%). Audiovisual feedback and star awards were seen as helpful (n=8, 40%), with a preference for more specific motivational feedback (n=9, 45%) and clearer performance summaries (n=8, 40%). Performance ratings should be simple and understandable (n=9, 45%), with varying levels to sustain motivation (n=6, 30%). The system’s innovative approach to addressing cognitive and physical functions was well-received (n=9, 45%), and therapists requested a wider range of customizable exercises to cater to individual patient needs (n=10, 50%). The safety harness was valued for creating secure training environments (n=4, 20%). For independent exercising, therapists suggested clearer, shorter activity instructions that remain visible until acknowledged (n=6, 30%), underscoring the importance of considering the patient’s cognitive and physical capabilities for independent use (n=16, 80%). Theme descriptions and quotes are available in Table S1 in Multimedia Appendix 7.

#### Both User Groups

PEUs and SEUs have identified opportunities to enhance system functionality and user experience. Key improvements include reducing inertia and swing of the swivel arm for better training comfort, making the safety system operable for therapists of all heights, and ensuring accurate tracking. Adjustments for color-blind users and projector light issues will enhance visual clarity. Clear instructional texts and synchronized movements will improve gameplay fluidity. Additionally, automatic screen arrangement correction, seamless activity transitions, and reliable HTC Vive tracker recognition will boost overall system performance.

## Discussion

### Principal Results and Comparison With Prior Work

Patients and therapists reported positive experiences and good usability with the ExerG exergame. PEUs deemed 79% of instructions as acceptable, achieved good exercise performance in 65% of tasks, and completed 97% of tasks without adverse events. Patients expressed satisfaction, with 88% reporting positive emotions and a median user satisfaction score of 9. Many patients indicated a willingness to continue using the device, contingent upon improvements in graphic design, tracking systems, projector quality, clarity of instructions, and game variety. PEUs felt secure in the safety harness, though some suggested enhancements for swivel arm movement. SEUs demonstrated effective task execution, with minimal hesitation reported. Therapist usability ratings were high, with all willing to use the ExerG and being able to operate it based on the user handbook. They noted the motivating effects of video-game-based training and its support for physical skills. Suggestions for improvement included incorporating daily living task simulations, more customization, targeted feedback, clearer performance ratings, and concise instructions.

This usability study with patients and therapists from 2 European rehabilitation centers identified specific usability challenges and strengths, informing design recommendations. Despite initial challenges inherent to the functional model, patients reported increased engagement and enjoyment across exercising rounds. Positive emotions, particularly heightened attention and enjoyment, characterized their experiences. While feedback on game mechanics was mostly favorable, some patients expressed a desire for greater autonomy. These findings underscore ExerG’s potential for user engagement and highlight key areas for refinement in future versions.

Our findings support the OPTIMAL (Optimizing Performance Through Intrinsic Motivation and Attention for Learning) theory of motor learning [38], which emphasizes the importance of learner autonomy in improving motor learning. Research shows that people are more motivated when they have control over their actions and their impact on the environment, even if it requires more effort. This indicates an inherent reward in exercising control. Eitam et al [39] demonstrated that motivation increases when individuals feel their actions have meaningful effects, underscoring the role of autonomy in fostering intrinsic motivation. Most patients had prior experience with rehabilitation technologies, but the exergame in this study was more complex due to its multisensory and multimodal features, unique movement concepts, and cognitive challenges. This complexity may have affected patients’ autonomy, particularly with in-game instructions that were always clear. However, exergames are known to promote autonomy and competence through playful engagement once users become familiar with the technology [40]. Patients reported an increase in perceived physical effort from the first to the second ExerG exercising round, while mental effort was perceived as lower. Overall, patients had a very good player experience, suggesting strong potential for ExerG training despite initial challenges.

In the quantitative assessment, semistructured interviews with PEUs showed that most patients enjoyed training with the ExerG software and had positive responses to its accessories, with no adverse events reported across study centers. SEUs found that the setup and closure of ExerG usage training and observation rounds were generally well-received. No adverse events were reported across study centers.

Potential optimizations for the ExerG system include reducing inertia and swing effects in the swivel arm for patient comfort, ensuring accurate tracking without offset, making accommodations for color-blind individuals, addressing projector light issues affecting color perception, and ensuring proper setup of ground patterns, room darkening, and fully charged trackers.

From a motor learning perspective, these adjustments are important for several reasons: First, ExerG training involves physical-cognitive dual-task training for patients with motor or cognitive disorders, where cognitive-motor interference leads to decreased performance [41]. Second, aging [42] and neurological disorders [43] increase susceptibility to this interference. Third, both aging [44] and neurological disorders [43,45] impair off-line gains in motor skill learning and heighten susceptibility to contextual interference. This interference occurs when practicing multiple skills in 1 session, with high contextual interference aiding retention and transfer despite lower immediate performance [44]. Therefore, minimizing external factors, such as lighting, noise, or the ExerG’s safety harness, is essential to optimize performance.

Older individuals are primarily motivated to engage with exergames by generativity, peer recommendations, self-improvement, and curiosity [46]. Retention is enhanced by achieving goals, immersion, and staying active, supported by structured schedules and adjustable difficulty levels. However, barriers such as stagnant progress, fatigue, or discomfort can hinder participation. Thus, it is essential to adapt the ExerG training software and accessories based on these psychological and user experience insights.

From an SEU perspective, clear instructions, accurate alignment of object movements with player actions, automatic screen arrangement, smooth transitions, easy tracker recognition and calibration, user-friendly safety systems, and prompt projector remote responses were highlighted. Our findings align with a systematic review indicating that ease-of-use, comfort, learnability, usefulness, and perceived efficacy are crucial usability aspects for health care professionals [47]. These insights underscore the need for close collaboration between the game design and development team and SEUs to incorporate SEUs’ domain knowledge into the game design. Overall, this study’s findings suggest that the ExerG training software and accessories can be further iterated and optimized to better meet patient and therapist requirements.

### Strengths and Limitations

This study adds to the literature on exergames for rehabilitation by delivering insights into the usability, safety, performance, and user experience of a novel exergame functional model in a real-world setting. Despite the challenges, the ExerG showed overall good usability and positive experiences for both patients and therapists. Patients reported increased enjoyment and improved performance, with 88% of their emotions being positive, while therapists successfully executed training scenarios and identified areas for optimization, including clearer instructions, more accurate tracking, and refined feedback mechanisms. Importantly, no adverse events occurred during this study, indicating the safety of the ExerG system.

Our research underscores the importance of an interdisciplinary, iterative development approach for creating user-centered designs in rehabilitation technologies. By involving game and industrial designers, programmers, rehabilitation experts, and end users throughout the process, we developed a functional model that meets specific rehabilitation needs. Insights from this usability study will guide future iterations of the ExerG system to better serve patients and therapists. This approach can serve as a model for developing other rehabilitation technologies, emphasizing the significance of user involvement, iterative design, and real-world testing. Our study also has some limitations. While 20 SEUs were recruited from 2 rehabilitation centers, only patients from 1 center participated, resulting in a smaller sample size of 15 patients. Saturation was achieved in the qualitative strand, and prior research suggests that even with 15 users, a detection rate of 97% (minimum 90) is feasible [48]. The PEU group was heterogeneous, reflecting diverse diagnoses and symptoms, which may increase variability in outcomes but also enriches feedback by providing a broader range of perspectives. This diversity enhances the ExerG’s applicability and improves this study’s ecological validity, aligning findings more closely with real-world usage scenarios.

One significant limitation of our study is that, although our eligibility criteria permitted the inclusion of patients with an MMSE score of 19 or greater, only 1 patient with mild cognitive impairment participated, and there were no participants with moderate cognitive impairment. This lack of diversity in cognitive impairment levels may restrict the generalizability of our findings. Furthermore, motor and balance impairments were assessed by neurologists at the start of rehabilitation for this study’s participants. This study’s population included 3 patients who required walking aids and 5 others with a history of falls. However, aside from the 2 Berg Balance Scale items, no objective balance assessments or formal motor function assessments were conducted. Additionally, the presence of researchers in our usability study may have influenced participants’ responses and behavior. While patients were unaware if their study therapist was a researcher, some may have felt compelled to provide positive feedback or altered their behavior due to the observers’ presence. Although we recognize the potential for observer bias, it is a common concern in usability studies. Finally, assessing the exergame in its functional model stage may have affected usability and experience. However, since the development process followed an iterative approach, the findings can directly inform design optimizations for the exergame.

### Conclusions

The newly developed exergame (ExerG training software and accessories) demonstrated good usability and positive effects on both therapists and patients, despite challenges related to its functional model status. The interdisciplinary, iterative approach used in its development facilitated direct optimization based on study findings. Future iterations hold promise, including follow-up studies such as randomized controlled trials to assess long-term training effects and user experience with the finalized ExerG system. In conclusion, our study offers a comprehensive evaluation of the usability and user experience of a novel exergame in a rehabilitation setting, enhancing the understanding of how exergames can be designed to support rehabilitation outcomes while identifying key challenges and improvement opportunities.

## Supplementary material

10.2196/66515 Multimedia Appendix 1 Good Reporting of a Mixed Methods Study (GRAMMS) checklist.

10.2196/66515 Multimedia Appendix 2 Study material–exercise and test item details.

10.2196/66515 Multimedia Appendix 3 Details on screening instruments and secondary outcome measures for primary end users.

10.2196/66515 Multimedia Appendix 4 Case report forms of primary and secondary end users.

10.2196/66515 Multimedia Appendix 5 Further illustration of outcomes in both user groups.

10.2196/66515 Multimedia Appendix 6 Primary end user theme description.

10.2196/66515 Multimedia Appendix 7 Secondary end user theme description.

## Abbreviations

CR10: Borg Category Ratio Scale
MMSE: Mini Mental State Examination
OPTIMAL: Optimizing Performance Through Intrinsic Motivation and Attention for Learning
PEU: primary end user
PXI: Player Experience Inventory
SEU: secondary end user
SUS: System Usability Scale
