# Correction: Exergame (ExerG)-Based Physical-Cognitive Training for Rehabilitation in Adults With Motor and Balance Impairments: Usability Study

**DOI:** 10.2196/73405

**Published:** 2025-04-04

**Authors:** Silvia Herren, Barbara Seebacher, Sarah Mildner, Yanick Riederer, Ulrike Pachmann, Nija Sonja Böckler, Stephan Niedecken, Sabrina Alicia Sgandurra, Leo Bonati, Isabella Hotz, Alexandra Schättin, Roman Jurt, Christian Brenneis, Katharina Lenfert, Frank Behrendt, Stefan Schmidlin, Lennart Nacke, Corina Schuster-Amft, Anna Lisa Martin-Niedecken

**Affiliations:** 1 Research Department, Reha Rheinfelden, Rheinfelden, Switzerland; 2 Department of Rehabilitation Science, Clinic for Rehabilitation Muenster, Gröben 700, Muenster, 6232, Austria, 43 533720004 ext 6205; 3 Clinical Department of Neurology, Medical University of Innsbruck, Innsbruck, Austria; 4 Karl Landsteiner Institute for Interdisciplinary Rehabilitation Research, Muenster, Austria; 5 Sphery Ltd, Zurich, Switzerland; 6 VASCage – Centre on Clinical Stroke Research, Innsbruck, Austria; 7 Department of Design, Institute for Design Research, Zurich University of the Arts, Zurich, Switzerland; 8 English Language and Literature, Faculty of Arts, University of Waterloo, Waterloo, ON, Canada; 9 Stroke Center and Department of Neurology, University Hospital Basel, Basel, Switzerland; 10 Department of Clinical Research, University of Basel, Basel, Switzerland; 11 Department of Neurology, Clinic for Rehabilitation Muenster, Muenster, Austria; 12 School of Engineering and Computer Science, Bern University of Applied Sciences, Burgdorf, Switzerland; 13 HCI Games Group, Stratford School of Interaction Design and Business, University of Waterloo, Waterloo, ON, Canada; 14 Department for Sport, Exercise and Health, University of Basel, Basel, Switzerland

In “Exergame (ExerG)-Based Physical-Cognitive Training for Rehabilitation in Adults With Motor and Balance Impairments: Usability Study” (JMIR Serious Games 2025;13:e66515) the authors noted one error.

The files attached to the manuscript as Multimedia Appendices 4 and 5 were mixed. To correct this, the PDF that was originally attached as:

Multimedia Appendix 4: Further illustration of outcomes in both user groups.

Has been changed to:

Multimedia Appendix 4: Case report forms of primary and secondary end users.

Furthermore, the PDF that was originally attached to the article as:

Multimedia Appendix 5: Case report forms of primary and secondary end users.

Has been changed to:

Multimedia Appendix 5: Further illustration of outcomes in both user groups.

These files have been attached to this correction notice, with their appropriate captions, as Multimedia Appendix 1 and Multimedia Appendix 2.

The correction will appear in the online version of the paper on the JMIR Publications website, together with the publication of this correction notice. Because this was made after submission to PubMed, PubMed Central, and other full-text repositories, the corrected article has also been resubmitted to those repositories.

## Supplementary material

10.2196/73405 Multimedia Appendix 1 Case report forms of primary and secondary end users.

10.2196/73405 Multimedia Appendix 2 Further illustration of outcomes in both user groups.

