# Supplementary material for: Exergame (ExerG)-Based Physical-Cognitive Training for Rehabilitation in Adults With Motor and Balance Impairments: Usability Study
Source: JMIR Serious Games. 2025 Feb 14;13:e66515. doi: 10.2196/66515 (PMC11844876; doi:10.2196/66515)
Supplement: Multimedia Appendix 1 [file games-v13-e66515-s001.pdf]

**Guideline**	**Section: page**
Describe the justification for using a mixed methods approach to the research question	Methods- Study Design: p. 5
Describe the design in terms of the purpose, priority and sequence of methods	Methods – Study Design: p. 5
Describe each method in terms of sampling, data collection and analysis	Study Flowchart: Figure 2<br>Patients and Participants: pp. 6-7<br>Study Procedures: p. 8<br>Data Collection: pp. 8-10<br>Multimedia Appendix: 2<br>Data Analyses: pp. 10-11
Describe where integration has occurred, how it has occurred and who has participated in it	Results – Both User Groups: pp. 20-21
Describe any limitation of one method associated with the present of the other method	Discussion – Strengths and Limitations: pp. 23-24
Describe any insights gained from mixing or integrating methods	Discussion and Conclusions: pp. 21-24

O'Cathain A, Murphy E, Nicholl J. The quality of mixed methods studies in health services research. J Health Serv Res Policy. 2008;13: 92-98
