# Supplementary material for: Exergame (ExerG)-Based Physical-Cognitive Training for Rehabilitation in Adults With Motor and Balance Impairments: Usability Study
Source: JMIR Serious Games. 2025 Feb 14;13:e66515. doi: 10.2196/66515 (PMC11844876; doi:10.2196/66515)
Supplement: Multimedia Appendix 2 [file games-v13-e66515-s002.pdf]

Exercise

The ExerG exercise program combines various physical-cognitive training elements such as balance, coordination, and dual-tasking through engaging mini-games like walking, apple picking, pattern matching, and rowing. Other activities include navigating tall grass, trunk jumping, avoiding nuts, time-limited pattern matching, catching balloons, and multitasking in apple picking. These exercises are designed to improve coordinative skills by training precise spatial and temporal movements of the legs and arms and enhancing the control and regulation of motor activity by the central nervous system. Additionally, they address conditional abilities by requiring dynamic balance during rapid leg movements to prevent falls.

The video game environment also aims to activate executive functions—including flexibility, inhibition, and working memory—and attentional functions, such as selective and divided attention. The ExerG integrates physical and cognitive training by engaging these functions simultaneously, promoting effective dual-tasking. The training protocol follows the FITT-VP principles[1], which include:

- **Frequency**: the number of sessions per week
- **Intensity**: varying difficulty levels of the exergame activities (easy, medium, hard)
- **Time**: duration of each training session
- **Type**: combined physical-cognitive training (exergame)
- **Variability**: a range of different activities within the exergame
- **Progression**: gradual increase in training frequency, intensity, and session duration

The variety of video games allows tailoring of exercises to individual physical and cognitive abilities. Therapists can customize settings before each session and make real-time adjustments to match the individual's needs. These adjustments include selecting intensity levels, choosing exercises, and setting session duration.

Training intensities range from easy to hard. For walking, intensities vary from lifting legs low at a self-paced speed to high lifting at a fast speed. Dual-task walking incorporates arm movements of increasing complexity. Side steps progress with arm movements and reactive responses to stimuli. Front steps and lunges range from small to large steps, while squat depths increase from light to deep bends. Jumps advance from simple arm raises to jumps breaking floor contact, and the swaying tree pose becomes more challenging with body sway.

Balance exercises range from short single leg stances to full leg lifts, with symmetrical and asymmetrical arm tasks, as well as body rotations and bending. Swimming arm movements speed up from slow to fast. Movement challenges involve reaching for wall stimuli with varying upper body rotations. Balloon-catching exercises vary by speed, and obstacle jumps progress from galloping to normal jumps. Fruit sorting speeds range from slow to fast.

Test Items

The ExerG comprises training software and hardware accessories, including a safety harness for the ExerCube. The ExerCube consists of three padded walls held by a steel construction, arranged into an open cube. The cube measures 3.5m (width, open end), 2.3m (width, front wall), 2.8m (height), and 2.6m (straight-line depth). Three beamers, mounted on the ceiling of the cube, project different virtual game environments onto the padded walls, which also serve as a haptic interface for the user. A customized whole-body motion tracking system via HTC Vive trackers (HTC Corporation, Taoyuan, Taiwan), worn on ankles and wrists during the training, captures the user’s movements and exercises performed in the cube setting and transmits them to the screen in real-time. The newly developed supporting material, made of

steel, was specifically evaluated by an independent test laboratory for use in rehabilitation centers.

References

1. Garber CE, Blissmer B, Deschenes MR, Franklin BA, Lamonte MJ, Lee IM, et al. American College of Sports Medicine position stand. Quantity and quality of exercise for developing and maintaining cardiorespiratory, musculoskeletal, and neuromotor fitness in apparently healthy adults: guidance for prescribing exercise. Med Sci Sports Exerc. 2011 Jul;43(7):1334-59. PMID: 21694556. doi: 10.1249/MSS.0b013e318213fefb.
