# Supplementary material for: Exergame (ExerG)-Based Physical-Cognitive Training for Rehabilitation in Adults With Motor and Balance Impairments: Usability Study
Source: JMIR Serious Games. 2025 Feb 14;13:e66515. doi: 10.2196/66515 (PMC11844876; doi:10.2196/66515)
Supplement: Multimedia Appendix 3 [file games-v13-e66515-s003.docx]

Screening Instruments

The Mini Mental State Examination (MMSE) is a standardized assessment used to evaluate cognitive function, including temporal and spatial orientation, memory and recall, attention, language and comprehension, as well as reading, writing, drawing, and arithmetic [1]. This test serves as a screening tool for clinical examinations and consists of various tasks and questions aimed at assessing cognitive abilities. A maximum score of 30 points is possible, with a cut-off score of 24 points being the most widely used to indicate dementia in elderly medical patients [2]. The MMSE typically takes about 7 to 10 minutes to complete [1]. In middle-aged and older adults, The MMSE demonstrated excellent inter- and intra-rater reliability, with an intraclass correlation coefficient (ICC) of 0.75 [3]. In a study of community-dwelling patients (n = 283), including individuals over 75 regardless of cognitive status and those aged 50-74 with memory issues but no depression or delirium, the MMSE showed adequate predictive validity for dementia, with an ROC curve area of 0.85 [4]. Another study including 299 patients with mild cognitive impairment (MCI) has demonstrated excellent convergent validity of the MMSE with the Montreal Cognitive Assessment (MOCA) (r = 0.60) [5]. Among 219 healthy controls, the correlation was adequate (r = 0.43). In a mixed population of 618 participants, which included 299 with MCI, 219 healthy controls, and 100 with dementia, the MMSE exhibited excellent convergent validity with the MOCA (r = 0.84). In a study involving 129 patients with Parkinson's disease (57% female), the MMSE showed a negative correlation with age (p < .001) and motor score B of the Unified Parkinson’s Disease Rating Scale (UPDRS) (p < .001) [6]. Additionally, there was a significant interaction between age and time (p < .01) as well as motor score B and time, indicating that cognitive decline accelerated with older age and higher motor scores. In a meta-analysis of mixed populations, adequate to excellent internal consistency of the MMSE was reported, with Cronbach's alpha ranging from 0.54 to 0.96 [7]. The construct validity of the MMSE is robust, evidenced by a significant correlation of 0.87 (p<0.001) with the Dementia Rating Scale (Aarsland et al., 2001), and it has been shown to effectively measure cognitive decline in patients with Parkinson’s disease over periods of approximately 10 years [8].

The Berg Balance Scale (BBS) is a widely used assessment tool in physiotherapy to evaluate the balance capabilities of patients [9]. It consists of 14 activities that a therapist observes and rates in terms of static and dynamic balance [9]. These activities are based on everyday actions, including transfers from sitting to standing, standing to sitting, and lateral movements; static balance tasks such as standing and sitting without support, standing with closed eyes, standing with feet positioned closely together, reaching forward with both arms, picking up an object from the floor, looking over the shoulder while standing, turning 360 degrees, tandem standing, and one-legged standing. Dynamic balance is assessed by having patients alternate placing their feet on a stepping stool. Each item is scored from 0 to 4, where 0 indicates that the patient requires maximal assistance to perform the task, and 4 signifies that the task can be completed without issues, resulting in a maximum possible score of 56 points. After scoring, patients are classified into three groups: a score of 0-20 suggests the need for a wheelchair, 21-40 indicates the need for walking assistance, and 41-56 reflects the ability to walk independently [9]. The BBS takes approximately 15 to 20 minutes to complete [9]. In a study involving 44 older adults, with a mean age of 74.6 (SD 5.4) years for non-fallers and 77.6 (SD 7.8) years for fallers, a history of falls along with a BBS score of < 51, or no history of falls with a BBS score of < 42, was predictive of falls, demonstrating 91% sensitivity and 82% specificity; additionally, a BBS score of < 40 was associated with nearly 100% fall risk [10]. Another study found a cutoff score of ≤52 out of 56 points to indicate an increased risk of falls (sensitivity=0.64, specificity=0.7067) in people with Parkinson’s disease [11]. In a study involving community-dwelling older adults, the Berg Balance Scale (BBS) demonstrated excellent intrarater reliability with an intraclass correlation coefficient (ICC) of 0.98, as well as excellent interrater reliability, also with an ICC of 0.98 [12]. In another study focusing on institutionalized older adults, the BBS again showed excellent intrarater reliability, with an ICC of 0.97 [13]. In people with Parkinson’s disease, excellent test-retest reliability and inter-rater reliability have been shown, with intraclass correlation coefficients (ICCs) of 0.80 and 0.95, respectively [14]. In a study involving individuals with chronic stroke, the Berg Balance Scale (BBS) showed an excellent correlation with the Dynamic Gait Index (DGI), with correlation coefficients of r = 0.75 for the first reading and r = 0.77 for the second reading. Additionally, there was an adequate correlation between the BBS and the Timed Up and Go (TUG) test, with coefficients of r = -0.52 for the first reading and r = -0.53 for the second reading [15].

Secondary Outcome Measures

The Borg CR10 is a numerical scale for assessing subjective perception of effort and exertion. Though it originally ranged from six to twenty—correlating with heart rate in beats per minute divided by ten—the study used the CR scale from zero (‘no exertion at all’) to ten (‘maximal exertion’) [16]. This scale was chosen for its straightforward verbal descriptions, making it easily accessible to laypeople.

The Paas MERS is simple, 9-item numerical rating scale ranging from one (‘very, very low mental effort”) to nine (‘very, very high mental effort”) [17]. It is recommended for use alongside performance assessments and has been widely used in numerous studies.

The validated German version [18] of the PXI [19] was administered after both training rounds to evaluate player experience. Developed to understand the impact of game designers' choices on players' experiences, the PXI features 30 items measuring ten domains, with three questions per domain. These domains cover psychosocial and functional aspects such as immersion, autonomy, progress feedback, and ease of control. Each question is scored on a 7-point Likert scale from strongly disagree (-3) to strongly agree (3).

A trained team member systematically observed and documented the patient's emotions during gameplay [20]. Any emotions or difficulties noted or reported by the patient during or after the training were recorded and analyzed to uncover their causes and explore potential solutions [20]. Utilized systematic observation was characterized by three main criteria: The observation process involved (1) an explicit aim to observe patient emotions with full attention, (2) a systematic approach that adhered to a structured observation plan outlining what, when, and how to observe and code behaviors, and (3) quality control measures to ensure acceptable data quality and sufficient reliability [21]. In this study, reliability in observing emotions was ensured by using video recordings of the sessions, allowing the observer to review the footage multiple times for consistent application of the criteria. Inter-session reliability was verified by conducting observations during Exercising Rounds 1 and 2 to assess consistency over time. Validity was established by comparing behavioral emotion indicators with patient self-reports, reflecting the individuals' subjective experiences [21].

**References**

1. Folstein MF, Folstein SE, McHugh PR. "Mini-mental state". A practical method for grading the cognitive state of patients for the clinician. J Psychiatr Res. 1975 Nov;12(3):189-98. PMID: 1202204.

2. Lopez MN, Charter RA, Mostafavi B, Nibut LP, Smith WE. Psychometric properties of the Folstein Mini-Mental State Examination. Assessment. 2005 Jun;12(2):137-44. PMID: 15914716. doi: 10.1177/1073191105275412.

3. Feeney J, Savva GM, O'Regan C, King-Kallimanis B, Cronin H, Kenny RA. Measurement Error, Reliability, and Minimum Detectable Change in the Mini-Mental State Examination, Montreal Cognitive Assessment, and Color Trails Test among Community Living Middle-Aged and Older Adults. J Alzheimers Dis. 2016 May 31;53(3):1107-14. PMID: 27258421. doi: 10.3233/jad-160248.

4. Brodaty H, Pond D, Kemp NM, Luscombe G, Harding L, Berman K, et al. The GPCOG: a new screening test for dementia designed for general practice. J Am Geriatr Soc. 2002 Mar;50(3):530-4. PMID: 11943052. doi: 10.1046/j.1532-5415.2002.50122.x.

5. Trzepacz PT, Hochstetler H, Wang S, Walker B, Saykin AJ. Relationship between the Montreal Cognitive Assessment and Mini-mental State Examination for assessment of mild cognitive impairment in older adults. BMC Geriatr. 2015 Sep 7;15:107. PMID: 26346644. doi: 10.1186/s12877-015-0103-3.

6. Aarsland D, Andersen K, Larsen JP, Perry R, Wentzel-Larsen T, Lolk A, et al. The rate of cognitive decline in Parkinson disease. Arch Neurol. 2004 Dec;61(12):1906-11. PMID: 15596611. doi: 10.1001/archneur.61.12.1906.

7. Tombaugh TN, McIntyre NJ. The mini-mental state examination: a comprehensive review. J Am Geriatr Soc. 1992 Sep;40(9):922-35. PMID: 1512391. doi: 10.1111/j.1532-5415.1992.tb01992.x.

8. Lessig S, Nie D, Xu R, Corey-Bloom J. Changes on brief cognitive instruments over time in Parkinson's disease. Mov Disord. 2012 Aug;27(9):1125-8. PMID: 22692724. doi: 10.1002/mds.25070.

9. Berg KO, Wood-Dauphinee SL, Williams JI, Maki B. Measuring balance in the elderly: validation of an instrument. Can J Public Health. 1992 Jul-Aug;83 Suppl 2:S7-11. PMID: 1468055.

10. Shumway-Cook A, Baldwin M, Polissar NL, Gruber W. Predicting the probability for falls in community-dwelling older adults. Phys Ther. 1997 Aug;77(8):812-9. PMID: 9256869. doi: 10.1093/ptj/77.8.812.

11. Schlenstedt C, Brombacher S, Hartwigsen G, Weisser B, Möller B, Deuschl G. Comparison of the Fullerton Advanced Balance Scale, Mini-BESTest, and Berg Balance Scale to Predict Falls in Parkinson Disease. Phys Ther. 2016 Apr;96(4):494-501. PMID: 26381806. doi: 10.2522/ptj.20150249.

12. Berg KO, Maki BE, Williams JI, Holliday PJ, Wood-Dauphinee SL. Clinical and laboratory measures of postural balance in an elderly population. Arch Phys Med Rehabil. 1992 Nov;73(11):1073-80. PMID: 1444775.

13. Conradsson M, Lundin-Olsson L, Lindelöf N, Littbrand H, Malmqvist L, Gustafson Y, et al. Berg balance scale: intrarater test-retest reliability among older people dependent in activities of daily living and living in residential care facilities. Phys Ther. 2007 Sep;87(9):1155-63. PMID: 17636155. doi: 10.2522/ptj.20060343.

14. Leddy AL, Crowner BE, Earhart GM. Functional gait assessment and balance evaluation system test: reliability, validity, sensitivity, and specificity for identifying individuals with Parkinson disease who fall. Phys Ther. 2011 Jan;91(1):102-13. PMID: 21071506. doi: 10.2522/ptj.20100113.

15. Alghadir AH, Al-Eisa ES, Anwer S, Sarkar B. Reliability, validity, and responsiveness of three scales for measuring balance in patients with chronic stroke. BMC Neurol. 2018 Sep 13;18(1):141. PMID: 30213258. doi: 10.1186/s12883-018-1146-9.

16. Borg GA. Psychophysical bases of perceived exertion. Med Sci Sports Exerc. 1982;14(5):377-81. PMID: 7154893.

17. Paas FGWC. Training strategies for attaining transfer of problem-solving skill in statistics: A cognitive-load approach. Journal of Educational Psychology. 1992;84(4):429–34. doi: 10.1037/0022-0663.84.4.429.

18. Graf L, Altmeyer M, Emmerich K, Herrlich M, Krekhov A, Spiel K. Development and Validation of a German Version of the Player Experience Inventory (PXI). Proceedings of Mensch und Computer 2022; Darmstadt, Germany: Association for Computing Machinery; 2022. p. 265–75.

19. Abeele VV, Spiel K, Nacke L, Johnson D, Gerling K. Development and validation of the player experience inventory: A scale to measure player experiences at the level of functional and psychosocial consequences. International Journal of Human-Computer Studies. 2020 2020/03/01/;135:102370. doi: <https://doi.org/10.1016/j.ijhcs.2019.102370>.

20. International handbook of emotions in education. Pekrun R, Linnenbrink-Garcia L, editors. New York, NY, US: Routledge/Taylor & Francis Group; 2014. x, 698-x, p. ISBN: 978-0-415-89501-9 (Hardcover); 978-0-415-89502-6 (Paperback); 978-0-203-14821-1 (PDF).

21. Reisenzein R, Junge M, Studtmann M, Huber O. Observational approaches to the measurement of emotions. International handbook of emotions in education. New York, NY, US: Routledge/Taylor & Francis Group; 2014. p. 580-606.
