# Supplementary material for: Exergame (ExerG)-Based Physical-Cognitive Training for Rehabilitation in Adults With Motor and Balance Impairments: Usability Study
Source: JMIR Serious Games. 2025 Feb 14;13:e66515. doi: 10.2196/66515 (PMC11844876; doi:10.2196/66515)
Supplement: Multimedia Appendix 4 [file games-v13-e66515-s004.pdf]

Primary end users

Patient ID:	
Written informed consent: Yes ☐ No ☐<br>*Explanation………………………..<br><br>** Please note: Individuals who do not have a signed informed consent form for the study must NOT be included in the study.*	
Date of the information: .... (MM/DD/YYYY)	
Study inclusion date: .... (MM/DD/YYYY)	
Declaration of informed consent<br>Date of the participant's signature... (MM/DD/YYYY)<br>Date of signature of the principal investigator .... (MM/DD/YYYY)<br>Date and version of the signed informed consent form:<br>Date: .... (MM/DD/YYYY) Version: V...	
A copy of the signed participant information will be handed out:	☐ Yes, date: ....<br>☐ No, explanation:.............................
Date Study measurement:<br>...	Start measurement (time): ....<br>End of measurement (time): ....
Investigator (initials):	
Signature of the principal investigator:	

PRE-SCREENING

**Inclusion criteria**			
E1	Aged ≥ 18 years	☐ Yes	☐ No*
E2	Undergoing inpatient or outpatient rehabilitation at RHF[a]	☐ Yes	☐ No
E3	Motor impairment of the upper or lower extremities, gait disorder, balance disorder, visual-spatial disorder, or cognitive disorder due to any disease	☐ Yes	☐ No
E4	Ability to speak and comprehend German and understand digitally transmitted training instructions	☐ Yes	☐ No
E5	No prior training experience with the device	☐ Yes	☐ No
E6	Body height ranging from 160 cm to 200 cm, in accordance with hardware specifications	☐ Y	☐ No

** Please note: If one of the criteria is answered with NO, the individual is not suitable for participation in the study and must therefore NOT be included.*

**Exclusion criteria**			
A1	Cognitive impairment as defined by a Mini Mental State Examination (MMSE) score of ≤18	☐ Yes *	☐ No
A2	Known cybersickness	☐ Yes	☐ No
A3	Severe visual, neurological, cardiorespiratory, psychiatric, or orthopedic impairments that reduce a person's ability to follow instructions or play the games	☐ Yes	☐ No
A4	Epileptic seizures within the past three months	☐ Yes	☐ No
A5	Recent surgery, fractures, joint replacement, or malignancy within the past three months	☐ Yes	☐ No
A6	Impairment of hearing resulting in the inability to engage in verbal communication	☐ Yes	☐ No
A7	Severe movement pain exceeding 5 on the 11-point pain Numeric Rating Scale	☐ Yes	☐ No
A8	Physical conditions that prevent the proper wearing of the safety harness or could result in pain or health complications, such as skin lesions or open wounds (e.g., severe osteoporosis)	☐ Yes	☐ No
A9	Joint contractures (e.g., in the shoulder, knee, or hip) that may result in limitations during end-user evaluation of the ExerCube	☐ Yes	☐ No
A10	Severe neurological conditions (e.g., severe epilepsy, advanced Parkinson's disease, condition after severe stroke)	☐ Yes	☐ No
A11	Severe psychiatric conditions (e.g., pronounced paranoid states, severe depression)	☐ Yes	☐ No
A12	Terminal illness (estimated life expectancy <12 months)	☐ Yes	☐ No

* *Please note: If one of the criteria is answered with YES, the individual is not suitable for participation in the study and must therefore NOT be included.*

A. PERSONAL DATA

Age in years ....

Size: .... **Weight**: ....

Gender ☐ Male ☐ Female ☐ ....

Assistive devices (e.g. walking stick, wheelchair) ☐ Yes☐ No If yes, which ones:

....

Falls within the last 6 months? ☐ Yes☐ No If yes, frequency of falls: ....

....

....

- ☐ 1x in 6 months
- ☐ up to 5x in 6 months
- ☐ more than 5 times in 6 months

The most recent fall, date: ………………………………

Reason for the fall: ....

Experience with **technology-based/device-supported** training ☐ Yes☐ No

Setting (multiple answers possible):

- ☐ Rehabilitation/clinic/institution
- ☐ Therapy (physiotherapy, occupational therapy, speech therapy, etc.)
- ☐ Private (also self-paying, at home)

☐ Other: ....

Technologies (multiple answers possible):

- ☐ Stationary weight-relieving systems (e.g. Float, Rysen, C-Mill)
- ☐ Mobile weight-relieving systems (e.g. Andago)
- ☐ Exoskeleton (e.g. Lokomat, ArmeoSpring)
- ☐ Exergames (e.g. Dividat Senso, Myro, DD System Elite Legpress, Wii, ReDance)
- ☐ Virtual reality (e.g. VR glasses, YouGrabber)
- ☐ Other:

B. SCREENING ASSESSMENTS

1) Numerical Rating scale (pain) *(Hawker et al, 2011)*

No pain	0	1	2	3	4	5	6	7	8	9	10	Strongest imaginable pain

2) Berg Balance Scale: Items 1 and 2 *(Berg et al, 1992; Scherfer, E., et al, 2006)*

Item no.	Description	Assessment	Value
1	From sitting to standing	– Patient is seated in a free standing, standard height chair (18-20 in) with arm rests<br>– Instructions: Please stand up, try not to use your hands for support<br>– Scoring:<br>4= able to stand without using hands, stabilizes independently<br>3= able to stand independently using hands<br>2= able to stand using hands after several tries<br>1= needs minimal aid to stand or to stabilize<br>0= needs moderate or maximal assist to stand	
2	Standing without support	– Patient is standing quietly with feet shoulder width apart on a solid surface<br>– Examiner has stopwatch in hand<br>– Instructions: Please stand for 2 minutes without holding on<br>– Scoring:<br>(If a person is able to stand 2 minutes unsupported, score full points for<br>sitting unsupported. Proceed to item #4)<br>4= able to stand safely for 2 minutes<br>3= able to stand 2 minutes with supervision<br>2= able to stand 30 seconds unsupported<br>1= needs several tries to stand 30 seconds unsupported<br>0= unable to stand 30 seconds unsupported	

3) Mini-Mental Status Examination *(modified from Folstein, M. F., Folstein, S. E., & McHugh, P. R., 1975)*

Total score: ………………….

C. TRAINING PROTOCOL

The training was conducted by:	Introduction to training:	Duration: ………. min
☐ 1 Therapist (ID ………)<br>☐ Investigator (initials)	Date: ………………<br>Time: ………………<br>Location: ………………	Initials of the person who carried out the training:<br>……………

1) Observation protocol: Training interaction Visit 1, round 1

No	Activity	Level	Implement-ation	Comprehen-sibility	Exercises	Training intensity	Difficul-ties
				The patient understands what he/she has to do during the activity and how he/she can control the activity.	The patient is able to perform the physical exercises to control the activity.	The training intensity was...	Comments of the test persons
	Calibration	☐ 1 simple<br>☐ 2 medium<br>☐ 3 heavy<br>☐ 4 N/A	☐ 1 yes<br>☐ 2 no<br>☐ 3 skipped<br>☐ 4 N/A	☐ 1 very good<br>☐ 2 good<br>☐ 3 part-part<br>☐ 4 poor<br>☐ 5 very poor	☐ 1 very good<br>☐ 2 good<br>☐ 3 part-part<br>☐ 4 poor<br>☐ 5 very poor	☐ 1 too heavy<br>☐ 2 heavy<br>☐ 3 optimal<br>☐ 4 light<br>☐ 5 too light<br>☐ 6 N/A	
	Walking	☐ 1 simple<br>☐ 2 medium<br>☐ 3 heavy<br>☐ 4 N/A	☐ 1 yes<br>☐ 2 no<br>☐ 3 skipped<br>☐ 4 N/A	☐ 1 very good<br>☐ 2 good<br>☐ 3 part-part<br>☐ 4 poor<br>☐ 5 very poor	☐ 1 very good<br>☐ 2 good<br>☐ 3 part-part<br>☐ 4 poor<br>☐ 5 very poor	☐ 1 too heavy<br>☐ 2 heavy<br>☐ 3 optimal<br>☐ 4 light<br>☐ 5 too light<br>☐ 6 N/A	
	Picking apples	☐ 1 simple<br>☐ 2 medium<br>☐ 3 heavy<br>☐ 4 N/A	☐ 1 yes<br>☐ 2 no<br>☐ 3 skipped<br>☐ 4 N/A	☐ 1 very good<br>☐ 2 good<br>☐ 3 part-part<br>☐ 4 poor<br>☐ 5 very poor	☐ 1 very good<br>☐ 2 good<br>☐ 3 part-part<br>☐ 4 poor<br>☐ 5 very poor	☐ 1 too heavy<br>☐ 2 heavy<br>☐ 3 optimal<br>☐ 4 light<br>☐ 5 too light<br>☐ 6 N/A	
	Walking	☐ 1 simple<br>☐ 2 medium<br>☐ 3 heavy<br>☐ 4 N/A	☐ 1 yes<br>☐ 2 no<br>☐ 3 skipped<br>☐ 4 N/A	☐ 1 very good<br>☐ 2 good<br>☐ 3 part-part<br>☐ 4 poor<br>☐ 5 very poor	☐ 1 very good<br>☐ 2 good<br>☐ 3 part-part<br>☐ 4 poor<br>☐ 5 very poor	☐ 1 too heavy<br>☐ 2 heavy<br>☐ 3 optimal<br>☐ 4 light<br>☐ 5 too light<br>☐ 6 N/A	

	Rowing	☐ 1 simple<br>☐ 2 medium<br>☐ 3 heavy<br>☐ 4 N/A	☐ 1 yes<br>☐ 2 no<br>☐ 3 skipped<br>☐ 4 N/A	☐ 1 very good<br>☐ 2 good<br>☐ 3 part-part<br>☐ 4 poor<br>☐ 5 very poor	☐ 1 very good<br>☐ 2 good<br>☐ 3 part-part<br>☐ 4 poor<br>☐ 5 very poor	☐ 1 too heavy<br>☐ 2 heavy<br>☐ 3 optimal<br>☐ 4 light<br>☐ 5 too light<br>☐ 6 N/A	
	Walking	☐ 1 simple<br>☐ 2 medium<br>☐ 3 heavy<br>☐ 4 N/A	☐ 1 yes<br>☐ 2 no<br>☐ 3 skipped<br>☐ 4 N/A	☐ 1 very good<br>☐ 2 good<br>☐ 3 part-part<br>☐ 4 poor<br>☐ 5 very poor	☐ 1 very good<br>☐ 2 good<br>☐ 3 part-part<br>☐ 4 poor<br>☐ 5 very poor	☐ 1 too heavy<br>☐ 2 heavy<br>☐ 3 optimal<br>☐ 4 light<br>☐ 5 too light<br>☐ 6 N/A	
	Pattern matching	☐ 1 simple<br>☐ 2 medium<br>☐ 3 heavy<br>☐ 4 N/A	☐ 1 yes<br>☐ 2 no<br>☐ 3 skipped<br>☐ 4 N/A	☐ 1 very good<br>☐ 2 good<br>☐ 3 part-part<br>☐ 4 poor<br>☐ 5 very poor	☐ 1 very good<br>☐ 2 good<br>☐ 3 part-part<br>☐ 4 poor<br>☐ 5 very poor	☐ 1 too heavy<br>☐ 2 heavy<br>☐ 3 optimal<br>☐ 4 light<br>☐ 5 too light<br>☐ 6 N/A	
	Walking	☐ 1 simple<br>☐ 2 medium<br>☐ 3 heavy<br>☐ 4 N/A	☐ 1 yes<br>☐ 2 no<br>☐ 3 skipped<br>☐ 4 N/A	☐ 1 very good<br>☐ 2 good<br>☐ 3 part-part<br>☐ 4 poor<br>☐ 5 very poor	☐ 1 very good<br>☐ 2 good<br>☐ 3 part-part<br>☐ 4 poor<br>☐ 5 very poor	☐ 1 too heavy<br>☐ 2 heavy<br>☐ 3 optimal<br>☐ 4 light<br>☐ 5 too light<br>☐ 6 N/A	
	Picking apples	☐ 1 simple<br>☐ 2 medium<br>☐ 3 heavy<br>☐ 4 N/A	☐ 1 yes<br>☐ 2 no<br>☐ 3 skipped<br>☐ 4 N/A	☐ 1 very good<br>☐ 2 good<br>☐ 3 part-part<br>☐ 4 poor<br>☐ 5 very poor	☐ 1 very good<br>☐ 2 good<br>☐ 3 part-part<br>☐ 4 poor<br>☐ 5 very poor	☐ 1 too heavy<br>☐ 2 heavy<br>☐ 3 optimal<br>☐ 4 light<br>☐ 5 too light<br>☐ 6 N/A	
	Walking	☐ 1 simple<br>☐ 2 medium<br>☐ 3 heavy<br>☐ 4 N/A	☐ 1 yes<br>☐ 2 no<br>☐ 3 skipped<br>☐ 4 N/A	☐ 1 very good<br>☐ 2 good<br>☐ 3 part-part<br>☐ 4 poor<br>☐ 5 very poor	☐ 1 very good<br>☐ 2 good<br>☐ 3 part-part<br>☐ 4 poor<br>☐ 5 very poor	☐ 1 too heavy<br>☐ 2 heavy<br>☐ 3 optimal<br>☐ 4 light<br>☐ 5 too light<br>☐ 6 N/A	
	Rowing	☐ 1 simple<br>☐ 2 medium<br>☐ 3 heavy<br>☐ 4 N/A	☐ 1 yes<br>☐ 2 no<br>☐ 3 skipped<br>☐ 4 N/A	☐ 1 very good<br>☐ 2 good<br>☐ 3 part-part<br>☐ 4 poor<br>☐ 5 very poor	☐ 1 very good<br>☐ 2 good<br>☐ 3 part-part<br>☐ 4 poor<br>☐ 5 very poor	☐ 1 too heavy<br>☐ 2 heavy<br>☐ 3 optimal<br>☐ 4 light<br>☐ 5 too light<br>☐ 6 N/A	
	Walking	☐ 1 simple<br>☐ 2 medium<br>☐ 3 heavy<br>☐ 4 N/A	☐ 1 yes<br>☐ 2 no<br>☐ 3 skipped<br>☐ 4 N/A	☐ 1 very good<br>☐ 2 good<br>☐ 3 part-part<br>☐ 4 poor<br>☐ 5 very poor	☐ 1 very good<br>☐ 2 good<br>☐ 3 part-part<br>☐ 4 poor<br>☐ 5 very poor	☐ 1 too heavy<br>☐ 2 heavy<br>☐ 3 optimal<br>☐ 4 light<br>☐ 5 too light<br>☐ 6 N/A	

	Pattern matching	☐ 1 simple<br>☐ 2 medium<br>☐ 3 heavy<br>☐ 4 N/A	☐ 1 yes<br>☐ 2 no<br>☐ 3 skipped<br>☐ 4 N/A	☐ 1 very good<br>☐ 2 good<br>☐ 3 part-part<br>☐ 4 poor<br>☐ 5 very poor	☐ 1 very good<br>☐ 2 good<br>☐ 3 part-part<br>☐ 4 poor<br>☐ 5 very poor	☐ 1 too heavy<br>☐ 2 heavy<br>☐ 3 optimal<br>☐ 4 light<br>☐ 5 too light<br>☐ 6 N/A	
	Walking	☐ 1 simple<br>☐ 2 medium<br>☐ 3 heavy<br>☐ 4 N/A	☐ 1 yes<br>☐ 2 no<br>☐ 3 skipped<br>☐ 4 N/A	☐ 1 very good<br>☐ 2 good<br>☐ 3 part-part<br>☐ 4 poor<br>☐ 5 very poor	☐ 1 very good<br>☐ 2 good<br>☐ 3 part-part<br>☐ 4 poor<br>☐ 5 very poor	☐ 1 too heavy<br>☐ 2 heavy<br>☐ 3 optimal<br>☐ 4 light<br>☐ 5 too light<br>☐ 6 N/A	

Total time: ___________

2. Observation protocol: Training interaction Visit 1, Round 2

**No.**	**Activity**	**Level**	**Implement-ation**	**Comprehen-sibility**	**Exercises**	**Training intensity**	**Diffi-culties**
				The patient understands what he/she has to do during the activity and how he/she can control the activity.	The patient is able to perform the physical exercises to control the activity.	The training intensity was...	(Comm-ents of the test persons)
	Calib-ration	☐ 1 simple<br>☐ 2 medium<br>☐ 3 heavy<br>☐ 4 N/A	☐ 1 yes<br>☐ 2 no<br>☐ 3 skipped<br>☐ 4 N/A	☐ 1 very good<br>☐ 2 good<br>☐ 3 part-part<br>☐ 4 poor<br>☐ 5 very poor	☐ 1 very good<br>☐ 2 good<br>☐ 3 part-part<br>☐ 4 poor<br>☐ 5 very poor	☐ 1 too heavy<br>☐ 2 heavy<br>☐ 3 optimal<br>☐ 4 light<br>☐ 5 too light<br>☐ 6 N/A	
	Walking	☐ 1 simple<br>☐ 2 medium<br>☐ 3 heavy<br>☐ 4 N/A	☐ 1 yes<br>☐ 2 no<br>☐ 3 skipped<br>☐ 4 N/A	☐ 1 very good<br>☐ 2 good<br>☐ 3 part-part<br>☐ 4 poor<br>☐ 5 very poor	☐ 1 very good<br>☐ 2 good<br>☐ 3 part-part<br>☐ 4 poor<br>☐ 5 very poor	☐ 1 too heavy<br>☐ 2 heavy<br>☐ 3 optimal<br>☐ 4 light<br>☐ 5 too light<br>☐ 6 N/A	
	…	☐ 1 simple<br>☐ 2 medium<br>☐ 3 heavy<br>☐ 4 N/A	☐ 1 yes<br>☐ 2 no<br>☐ 3 skipped<br>☐ 4 N/A	☐ 1 very good<br>☐ 2 good<br>☐ 3 part-part<br>☐ 4 poor<br>☐ 5 very poor	☐ 1 very good<br>☐ 2 good<br>☐ 3 part-part<br>☐ 4 poor<br>☐ 5 very poor	☐ 1 too heavy<br>☐ 2 heavy<br>☐ 3 optimal<br>☐ 4 light<br>☐ 5 too light<br>☐ 6 N/A	
	Walking	☐ 1 simple<br>☐ 2 medium<br>☐ 3 heavy<br>☐ 4 N/A	☐ 1 yes<br>☐ 2 no<br>☐ 3 skipped<br>☐ 4 N/A	☐ 1 very good<br>☐ 2 good<br>☐ 3 part-part<br>☐ 4 poor<br>☐ 5 very poor	☐ 1 very good<br>☐ 2 good<br>☐ 3 part-part<br>☐ 4 poor<br>☐ 5 very poor	☐ 1 too heavy<br>☐ 2 heavy<br>☐ 3 optimal<br>☐ 4 light<br>☐ 5 too light<br>☐ 6 N/A	
	…	☐ 1 simple<br>☐ 2 medium<br>☐ 3 heavy<br>☐ 4 N/A	☐ 1 yes<br>☐ 2 no<br>☐ 3 skipped<br>☐ 4 N/A	☐ 1 very good<br>☐ 2 good<br>☐ 3 part-part<br>☐ 4 poor<br>☐ 5 very poor	☐ 1 very good<br>☐ 2 good<br>☐ 3 part-part<br>☐ 4 poor<br>☐ 5 very poor	☐ 1 too heavy<br>☐ 2 heavy<br>☐ 3 optimal<br>☐ 4 light<br>☐ 5 too light<br>☐ 6 N/A	
	Walking	☐ 1 simple<br>☐ 2 medium<br>☐ 3 heavy<br>☐ 4 N/A	☐ 1 yes<br>☐ 2 no<br>☐ 3 skipped<br>☐ 4 N/A	☐ 1 very good<br>☐ 2 good<br>☐ 3 part-part<br>☐ 4 poor<br>☐ 5 very poor	☐ 1 very good<br>☐ 2 good<br>☐ 3 part-part<br>☐ 4 poor<br>☐ 5 very poor	☐ 1 too heavy<br>☐ 2 heavy<br>☐ 3 optimal<br>☐ 4 light<br>☐ 5 too light<br>☐ 6 N/A	

	…	☐ 1 simple<br>☐ 2 medium<br>☐ 3 heavy<br>☐ 4 N/A	☐ 1 yes<br>☐ 2 no<br>☐ 3 skipped<br>☐ 4 N/A	☐ 1 very good<br>☐ 2 good<br>☐ 3 part-part<br>☐ 4 poor<br>☐ 5 very poor	☐ 1 very good<br>☐ 2 good<br>☐ 3 part-part<br>☐ 4 poor<br>☐ 5 very poor	☐ 1 too heavy<br>☐ 2 heavy<br>☐ 3 optimal<br>☐ 4 light<br>☐ 5 too light	
	Walking	☐ 1 simple<br>☐ 2 medium<br>☐ 3 heavy<br>☐ 4 N/A	☐ 1 yes<br>☐ 2 no<br>☐ 3 skipped<br>☐ 4 N/A	☐ 1 very good<br>☐ 2 good<br>☐ 3 part-part<br>☐ 4 poor<br>☐ 5 very poor	☐ 1 very good<br>☐ 2 good<br>☐ 3 part-part<br>☐ 4 poor<br>☐ 5 very poor	☐ 1 too heavy<br>☐ 2 heavy<br>☐ 3 optimal<br>☐ 4 light<br>☐ 5 too light<br>☐ 6 N/A	
	…	☐ 1 simple<br>☐ 2 medium<br>☐ 3 heavy<br>☐ 4 N/A	☐ 1 yes<br>☐ 2 no<br>☐ 3 skipped<br>☐ 4 N/A	☐ 1 very good<br>☐ 2 good<br>☐ 3 part-part<br>☐ 4 poor<br>☐ 5 very poor	☐ 1 very good<br>☐ 2 good<br>☐ 3 part-part<br>☐ 4 poor<br>☐ 5 very poor	☐ 1 too heavy<br>☐ 2 heavy<br>☐ 3 optimal<br>☐ 4 light<br>☐ 5 too light<br>☐ 6 N/A	
	Walking	☐ 1 simple<br>☐ 2 medium<br>☐ 3 heavy<br>☐ 4 N/A	☐ 1 yes<br>☐ 2 no<br>☐ 3 skipped<br>☐ 4 N/A	☐ 1 very good<br>☐ 2 good<br>☐ 3 part-part<br>☐ 4 poor<br>☐ 5 very poor	☐ 1 very good<br>☐ 2 good<br>☐ 3 part-part<br>☐ 4 poor<br>☐ 5 very poor	☐ 1 too heavy<br>☐ 2 heavy<br>☐ 3 optimal<br>☐ 4 light<br>☐ 5 too light<br>☐ 6 N/A	
	…	☐ 1 simple<br>☐ 2 medium<br>☐ 3 heavy<br>☐ 4 N/A	☐ 1 yes<br>☐ 2 no<br>☐ 3 skipped<br>☐ 4 N/A	☐ 1 very good<br>☐ 2 good<br>☐ 3 part-part<br>☐ 4 poor<br>☐ 5 very poor	☐ 1 very good<br>☐ 2 good<br>☐ 3 part-part<br>☐ 4 poor<br>☐ 5 very poor	☐ 1 too heavy<br>☐ 2 heavy<br>☐ 3 optimal<br>☐ 4 light<br>☐ 5 too light<br>☐ 6 N/A	
	Walking	☐ 1 simple<br>☐ 2 medium<br>☐ 3 heavy<br>☐ 4 N/A	☐ 1 yes<br>☐ 2 no<br>☐ 3 skipped<br>☐ 4 N/A	☐ 1 very good<br>☐ 2 good<br>☐ 3 part-part<br>☐ 4 poor<br>☐ 5 very poor	☐ 1 very good<br>☐ 2 good<br>☐ 3 part-part<br>☐ 4 poor<br>☐ 5 very poor	☐ 1 too heavy<br>☐ 2 heavy<br>☐ 3 optimal<br>☐ 4 light<br>☐ 5 too light<br>☐ 6 N/A	
	…	☐ 1 simple<br>☐ 2 medium<br>☐ 3 heavy<br>☐ 4 N/A	☐ 1 yes<br>☐ 2 no<br>☐ 3 skipped<br>☐ 4 N/A	☐ 1 very good<br>☐ 2 good<br>☐ 3 part-part<br>☐ 4 poor<br>☐ 5 very poor	☐ 1 very good<br>☐ 2 good<br>☐ 3 part-part<br>☐ 4 poor<br>☐ 5 very poor	☐ 1 too heavy<br>☐ 2 heavy<br>☐ 3 optimal<br>☐ 4 light<br>☐ 5 too light<br>☐ 6 N/A	
	Walking	☐ 1 simple<br>☐ 2 medium<br>☐ 3 heavy<br>☐ 4 N/A	☐ 1 yes<br>☐ 2 no<br>☐ 3 skipped<br>☐ 4 N/A	☐ 1 very good<br>☐ 2 good<br>☐ 3 part-part<br>☐ 4 poor<br>☐ 5 very poor	☐ 1 very good<br>☐ 2 good<br>☐ 3 part-part<br>☐ 4 poor<br>☐ 5 very poor	☐ 1 too heavy<br>☐ 2 heavy<br>☐ 3 optimal<br>☐ 4 light<br>☐ 5 too light<br>☐ 6 N/A	

Total time: ____________

3. Observation protocol: Training Interaction Visit 2, Rounds 1 & 2

Aspects	Rating round 1	Rating round 2
**Feedback system**<br><br>The feedback system of the videogame was helpful/supportive for the patient during implementation.	☐ 1 very good<br>☐ 2 good<br>☐ 3 part-part<br>☐ 4 poor<br>☐ 5 very poor	☐ 1 very good<br>☐ 2 good<br>☐ 3 part-part<br>☐ 4 poor<br>☐ 5 very poor
**Emotions during the training**<br><br>The following emotions were observed in the patient during the training:<br><br>*(multiple answers possible, brief explanation in the right-hand column)*	☐ 1 Joy<br>☐ 2 Curiosity<br>☐ 2 Attention<br>☐ 3 Surprise / amazement<br>☐ 4 Nervousness<br>☐ 4 Boredom<br>☐ 2 Confusion<br>☐ 4 Rejection<br>☐ 5 Anger	☐ 1 Joy<br>☐ 2 Curiosity<br>☐ 2 Attention<br>☐ 3 Surprise / amazement<br>☐ 4 Nervousness<br>☐ 4 Boredom<br>☐ 2 Confusion<br>☐ 4 Rejection<br>☐ 5 Anger

Observation protocol interview

Difficulties with the video game

- Asking about difficulties in training and/or incorrect performance of activities? What was the reason for the difficulties?
- Apart from the difficulties already mentioned, were there any other difficulties that were not observed?
- What would have helped to avoid the difficulty?

	**Observed**	**Problem/task (description)**
	☐ 1 Yes<br>☐ 2 No	
	☐ 1 Yes<br>☐ 2 No	
	☐ 1 Yes<br>☐ 2 No	
	☐ 1 Yes<br>☐ 2 No	
	☐ 1 Yes<br>☐ 2 No	
	☐ 1 Yes<br>☐ 2 No	
	☐ 1 Yes<br>☐ 2 No	
	☐ 1 Yes<br>☐ 2 No	
	☐ 1 Yes<br>☐ 2 No	
	☐ 1 Yes<br>☐ 2 No	

	☐ 1 Yes<br>☐ 2 No	
	☐ 1 Yes<br>☐ 2 No	
	☐ 1 Yes<br>☐ 2 No	
	☐ 1 Yes<br>☐ 2 No	
	☐ 1 Yes<br>☐ 2 No	
	☐ 1 Yes<br>☐ 2 No	
	☐ 1 Yes<br>☐ 2 No	
	☐ 1 Yes<br>☐ 2 No	
	☐ 1 Yes<br>☐ 2 No	
	☐ 1 Yes<br>☐ 2 No	
	☐ 1 Yes<br>☐ 2 No	
	☐ 1 Yes<br>☐ 2 No	
	☐ 1 Yes<br>☐ 2 No	

Scales and Questionnaires

1) Borg Category Ratio (CR)-10 Scale

(modified from Williams, 2017)

Score	Degree of physical exertion	Session 1 Score
0	Not exhausting at all	
0.5	very, very light	
1	very light	
2	light	
3	moderate	
4	somewhat strenuous	
5	exhausting	
6		
7	very strenuous	
8		
9	Very, very strenuous (almost maximum)	
10	Maximum effort	

- Round 1 BORG-CR10 Score: _______
- Round 2 BORG-CR10 Score: _______

2) Paas Mental Effort Scale

Paas Mental Effort Scale (modified from Martin et al., 2019)

**Score**	**Level of effort**	Session 1 Score
**1**	Very, very low mental effort //<br>Very, very little mental effort	
**2**		
**3**		
**4**		
**5**	Neither high nor low mental effort //<br>Neither low nor high mental effort	
**6**		
**7**		
**8**		
**9**	Very, very high mental effort //<br>Very, very high mental effort	

- Round 1 Paas Mental Effort Score: _______
- Round 2 Paas Mental Effort Score: _______

3) **Player Experience Inventory (PXI)** *(modified according to Abeele et al., 2020)*

Now please think about the game you played earlier. In particular, think about how you felt during the game. For each of the following statements, indicate the extent to which you agree with it.

**Item**	**Strongly disagree**	→					**Strongly agree**
	**-3**	**-2**	**-1**	**0**	**1**	**2**	**3**
1. Playing the game was meaningful to me.	☐	☐	☐	☐	☐	☐	☐
2 The game felt relevant to me.	☐	☐	☐	☐	☐	☐	☐
3. Playing this game was valuable to me.	☐	☐	☐	☐	☐	☐	☐
4. I wanted to explore how the game evolved.	☐	☐	☐	☐	☐	☐	☐
5. I wanted to find out how the game progressed.	☐	☐	☐	☐	☐	☐	☐
6. I felt eager to discover how the game continued.	☐	☐	☐	☐	☐	☐	☐
7. I felt I was good at playing this game.	☐	☐	☐	☐	☐	☐	☐
8. I felt capable while playing the game.	☐	☐	☐	☐	☐	☐	☐
9. I felt a sense of mastery playing this game.	☐	☐	☐	☐	☐	☐	☐
10 I felt free to play the game in my own way.	☐	☐	☐	☐	☐	☐	☐
11 I felt like I had choices regarding how I wanted to play this game.	☐	☐	☐	☐	☐	☐	☐
12 I felt a sense of freedom about how I wanted to play this game.	☐	☐	☐	☐	☐	☐	☐
13 I was no longer aware of my surroundings while I was playing.	☐	☐	☐	☐	☐	☐	☐
14 I was immersed in the game.	☐	☐	☐	☐	☐	☐	☐
15 I was fully focused on the game.	☐	☐	☐	☐	☐	☐	☐
16 The game informed me of my progress in the game.	☐	☐	☐	☐	☐	☐	☐

**Item**	**Strongly disagree**	→					**Strongly agree**
	**-3**	**-2**	**-1**	**0**	**-3**	**-2**	**-1**
17 I could easily assess how I was performing in the game.	☐	☐	☐	☐	☐	☐	☐
18 The game gave clear feedback on my progress towards the goals.	☐	☐	☐	☐	☐	☐	☐
19 I enjoyed the way the game was styled.	☐	☐	☐	☐	☐	☐	☐
20 I liked the look and feel of the game.	☐	☐	☐	☐	☐	☐	☐
21 I appreciated the aesthetics of the game.	☐	☐	☐	☐	☐	☐	☐
22 The game was not too easy and not too hard to play.	☐	☐	☐	☐	☐	☐	☐
23 The game was challenging but not too challenging.	☐	☐	☐	☐	☐	☐	☐
24 The challenges in the game were at the right level of difficulty for me.	☐	☐	☐	☐	☐	☐	☐
25 It was easy to know how to perform actions in the game.	☐	☐	☐	☐	☐	☐	☐
26 The actions to control the game were clear to me.	☐	☐	☐	☐	☐	☐	☐
27 I thought the game was easy to control.	☐	☐	☐	☐	☐	☐	☐
28 I grasped the overall goal of the game.	☐	☐	☐	☐	☐	☐	☐
29 The goals of the game were clear to me.	☐	☐	☐	☐	☐	☐	☐
30 I understood the objectives of the game.	☐	☐	☐	☐	☐	☐	☐

Semi-structured interview about the system interaction

Was the semi-structured interview conducted?

☐ 1 Yes ☐2 No → Explanation: ______________________________

No.	Question	Responses
1	On a scale from 1= very dissatisfied to 10= very satisfied, how satisfied were you with the use of the system?	……………… *(score from 1-10)*
2	What were your feelings about training with the system; did you find it enjoyable?	☐ 1 Yes<br>☐ 2 No, why *(brief explanation)*:………………………….
3	Did you understand the video game, or did you know what you had to do?	☐ 1 Yes<br>☐ 1 Yes, with (verbal) support from outside<br>☐ 2 No, why *(brief explanation)*:…………………………
4	Would you like to train with the system in your therapies?	☐ 1 Yes<br>☐ 2 No, why *(brief explanation)*:………………………..
5	Do you find that the feedback in the video game was helpful/supportive in the implementation?	☐ 1 Yes<br>☐ 2 Partial<br>☐ 3 No, why not *(brief explanation)*:…………………….<br>☐ 4 I did not notice
6	Did you feel safe during training?	☐ 1 Yes<br>☐ 2 Partial<br>☐ 3 No, why *(brief explanation)*:…………………………..
7	Did the safety system give you a feeling of security?	☐ 1 Yes<br>☐ 2 Partial<br>☐ 3 No, why *(brief explanation)*:
8	Were there any moments when you didn't feel safe?	☐ 1 Yes (short justification):<br>☐ 2 Partially *(brief explanation):*<br>…………………….<br>☐ 3 No
9	Did the safety system provide you with some freedom of movement?	☐ 1 Yes<br>☐ 2 Partial<br>☐ 3 No, why *(brief explanation)*:<br>………………

10	Was the safety system or harness comfortable to wear?	☐ 1 Yes<br>☐ 2 Partial<br>☐ 3 No, why *(brief explanation)*:<br>………………….

(Serious) Adverse Events

No.	Description	Type	Causal relationship
		☐ 1 AE<br>☐ 2 SAE*	☐ 1 Yes<br>☐ 2 No
		☐ 1 AE<br>☐ 2 SAE*	☐ 1 Yes<br>☐ 2 No
		☐ 1 AE<br>☐ 2 SAE*	☐ 1 Yes<br>☐ 2 No
		☐ 1 AE<br>☐ 2 SAE*	☐ 1 Yes<br>☐ 2 No
		☐ 1 AE<br>☐ 2 SAE*	☐ 1 Yes<br>☐ 2 No
		☐ 1 AE<br>☐ 2 SAE*	☐ 1 Yes<br>☐ 2 No
		☐ 1 AE<br>☐ 2 SAE*	☐ 1 Yes<br>☐ 2 No

**Any AE that results in death, is immediately life-threatening, results in hospitalization or prolongation of existing hospitalization, results in a congenital anomaly or birth defect, or results in permanent or severe disability or incapacity*

Device deficiencies

No	Type	Short description	Procedure	Led to SAE	Unexpected
	☐ 1 User interface<br>☐ 2 Training settings<br>☐ 3 Training implementation<br>☐ 4 Computer<br>☐ 5 HTC Vive Tracker<br>☐ 6 HTC Vive camera<br>☐ 7 Projector<br>☐ 8 Computer<br>☐ 9 Security system<br>☐ 10 Other:________________		Could it be fixed?<br>☐ 1 Yes<br>☐ 2 Yes, with user handbook<br>☐ 3 No<br>If no,<br>☐ 1 Cancellation of the visit	☐ 1 Yes<br>☐ 2 No	☐ 1 Yes<br>☐ 2 No
	☐ 1 User interface<br>☐ 2 Training settings<br>☐ 3 Training implementation<br>☐ 4 Computer<br>☐ 5 HTC Vive Tracker<br>☐ 6 HTC Vive camera<br>☐ 7 Projector<br>☐ 8 Computer<br>☐ 9 Security system<br>☐ 10 Other:_		Could it be fixed?<br>☐ 1 Yes<br>☐ 2 Yes, with user handbook<br>☐ 3 No<br>If no,<br>1 Cancellation of the visit	☐ 1 Yes<br>2 No	☐ 1 Yes<br>2 No
	☐ 1 User interface<br>☐ 2 Training settings<br>☐ 3 Training implementation<br>☐ 4 Computer<br>☐ 5 HTC Vive Tracker<br>☐ 6 HTC Vive camera<br>☐ 7 Projector<br>☐ 8 Computer<br>☐ 9 Security system<br>☐ 10 Other:_		Could it be fixed?<br>☐ 1 Yes<br>☐ 2 Yes, with user handbook<br>☐ 3 No<br>If no,<br>1 Cancellation of the visit	☐ 1 Yes<br>2 No	☐ 1 Yes<br>2 No
	☐ 1 User interface<br>☐ 2 Training settings<br>☐ 3 Training implementation<br>☐ 4 Computer<br>☐ 5 HTC Vive Tracker<br>☐ 6 HTC Vive camera<br>☐ 7 Projector<br>☐ 8 Computer<br>☐ 9 Security system<br>☐ 10 Other:_		Could it be fixed?<br>☐ 1 Yes<br>☐ 2 Yes, with user handbook<br>☐ 3 No<br>If no,<br>1 Cancellation of the visit	☐ 1 Yes<br>2 No	☐ 1 Yes<br>2 No

	☐ 1 User interface<br>☐ 2 Training settings<br>☐ 3 Training implementation<br>☐ 4 Computer<br>☐ 5 HTC Vive Tracker<br>☐ 6 HTC Vive camera<br>☐ 7 Projector<br>☐ 8 Computer<br>☐ 9 Security system<br>☐ 10 Other:_		Could it be fixed?<br>☐ 1 Yes<br>☐ 2 Yes, with user handbook<br>☐ 3 No<br>If no,<br>1 Cancellation of the visit	☐ 1 Yes<br>2 No	☐ 1 Yes<br>2 No
	☐ 1 User interface<br>☐ 2 Training settings<br>☐ 3 Training implementation<br>☐ 4 Computer<br>☐ 5 HTC Vive Tracker<br>☐ 6 HTC Vive camera<br>☐ 7 Projector<br>☐ 8 Computer<br>☐ 9 Security system<br>☐ 10 Other:_		Could it be fixed?<br>☐ 1 Yes<br>☐ 2 Yes, with user handbook<br>☐ 3 No<br>If no,<br>1 Cancellation of the visit	☐ 1 Yes<br>2 No	☐ 1 Yes<br>2 No
	☐ 1 User interface<br>☐ 2 Training settings<br>☐ 3 Training implementation<br>☐ 4 Computer<br>☐ 5 HTC Vive Tracker<br>☐ 6 HTC Vive camera<br>☐ 7 Projector<br>☐ 8 Computer<br>☐ 9 Security system<br>☐ 10 Other:_		Could it be fixed?<br>☐ 1 Yes<br>☐ 2 Yes, with user handbook<br>☐ 3 No<br>If no,<br>1 Cancellation of the visit	☐ 1 Yes<br>2 No	☐ 1 Yes<br>2 No
	☐ 1 User interface<br>☐ 2 Training settings<br>☐ 3 Training implementation<br>☐ 4 Computer<br>☐ 5 HTC Vive Tracker<br>☐ 6 HTC Vive camera<br>☐ 7 Projector<br>☐ 8 Computer<br>☐ 9 Security system<br>☐ 10 Other:_		Could it be fixed?<br>☐ 1 Yes<br>☐ 2 Yes, with user handbook<br>☐ 3 No<br>If no,<br>1 Cancellation of the visit	☐ 1 Yes<br>2 No	☐ 1 Yes<br>2 No

FINAL DOCUMENTATION

Date: ________________

(MM/DD/YYYY)

Visits performed:

☐ 1 Visit 1

☐ 2 Visit 2

Reason for ending the study:

☐ 1 Scheduled completion of the study according to the study protocol

☐ 2 Withdrawal of consent

☐ 3 Premature termination of the study by the sponsor

☐ 4 Other reason for early termination:

____

REFERENCES

1. Scherfer, E., Bohls, C., Freiberger, E., Heise, K.-F., Hogan, D. (2006). Berg-Balance-Scale - German Version - Translation of a Standardized Instrument for the Assessment of Balance and Risk of Falling. *Physioscience*, 2(2), 59-66.

2. Folstein, M. F., Folstein, S. E., & McHugh, P. R. (1975). "Mini-mental state": a practical method for grading the cognitive state of patients for the clinician. *Journal of psychiatric research*, *12*(3), 189-198.

3. Williams, N . (2017). The Borg rating of perceived exertion (RPE) scale. *Occupational Medicine*, *67*(5), 404-405.

4. Martin, L. J., Turnquist, A., Groot, B., Huang, S. Y., Kok, E., Thoma, B., & van Merriënboer, J. J. (2019). Exploring the role of infographics for summarizing medical literature. *Health Professions Education*, *5* (1), 48-57.

5. Borg GA. Psychophysical bases of perceived exertion. Med Sci Sports Exerc. 1982;14(5):377-81. PMID: 7154893.

6. Paas FGWC. Training strategies for attaining transfer of problem-solving skill in statistics: A cognitive-load approach. Journal of Educational Psychology. 1992;84(4):429–34. doi: 10.1037/0022-0663.84.4.429.

7. Abeele VV, Spiel K, Nacke L, Johnson D, Gerling K. Development and validation of the player experience inventory: A scale to measure player experiences at the level of functional and psychosocial consequences. International Journal of Human-Computer Studies. 2020 2020/03/01/;135:102370. doi: https://doi.org/10.1016/j.ijhcs.2019.102370.

Case Report Form
Secondary end users

Usability test protocol

ExerCube training software license

v5 Date: May 04.2023

Participant ID: S ___ ___

Table of contents

Study inclusion **3**
Information and declaration of consent 3
Review of the inclusion criteria 4
Demographic data 5
Training protocol **6**
Observation protocol **7**
Preparation of the hardware (Use scenario 1) 7
Preparation & attachment of the safety system (Use scenario 2)10
Simulation of a fall scenario (Use scenario 3) 12
Starting a predefined training program (Use scenario 4)13
During the training program15
Compilation of a predefined training sequence (Use scenario 5)16
Ending a training program (Use scenario 6) 19
Acceptance of the security system (Use scenario 7) 20
Terminating the hardware (Use scenario 8) 22
Procedure for specific situations with system problems or risks (knowledge tasks)23
Observation protocol interview29
Difficulties with the system29
Semi-structured interview / questionnaire **32**
Interaction with the system 32
System Usability Scale 35
Device deficiencies **36**
Final documentation **38**

Study inclusion

Date: __________ (MM/DD/YYYY)

Participant information and obtaining informed consent

Consent
Written informed consent for the study has been obtained:

1 ☐Yes ☐2 No*, → Explanation: ____

** Please note: Individuals who do not have a signed informed consent form for the study must NOT be included in the study.*

Participant information

Date of participant information: __________ (mm/dd/yyyy)

Declaration of consent

Date of signature of the participant: __________ (MM/DD/YYYY)

Date of signature/of the assessor: __________ (MM/DD/YYYY)

Date/version of the signed participant information and informed consent form:

__________ (MM/DD/YYYY) Version: V__ . __

Has the participant been given a copy of the signed participant information and informed consent form?

1 ☐Yes ☐2 No → Explanation: ____

Checking the inclusion criteria

Inclusion criteria:

	Yes	No*
**Age:** 21 years or older	☐1	☐2
**Professional group**: Sports scientists, training therapists or physiotherapists/occupational therapists specializing in sports/training therapy	☐1	☐2
**Professional qualification:** Completed training as a therapist or completed training as a scientist with a Bachelor's degree	☐1	☐2

** Please note: If one of the criteria is answered with NO, the subject is not suitable for participation in the study and must therefore NOT be included.*

Demographic data

Age in years: __________

Gender

1 ☐Male

2 ☐Female

3 ☐__________

Education in years: __________

Professional group:

1 ☐Physiotherapy

2 ☐Occupational therapy

3 ☐Sports training therapy

4 ☐Sports science

5 ☐Other: __________

Professional qualification (multiple answers possible):

1 ☐Vocational training/ higher technical college

2 ☐Bachelor

3 ☐Master's Degree/Diploma in Engineering

4 ☐Doctorate (PhD)

Department(s) (multiple answers possible):

1 ☐Neurology

2 ☐Orthopedics

3 ☐Geriatrics

4 ☐Medical Fitness

5 ☐Internal oncological rehabilitation

6 ☐Psychomotor rehabilitation

7 ☐Other: __________

Experience with technology-based/device-supported training
1 ☐Yes
2 ☐No

In which setting were the experiences made (multiple answers possible):
1 ☐Rehabilitation (inpatient setting)
2 ☐Rehabilitation (outpatient setting)
3 ☐Private (also self-pay, home setting)
4 ☐Other: __________

Technologies (multiple answers possible):
1 ☐Stationary weight-relieving systems (e.g. Float, Rysen, C-Mill)
2 ☐Mobile weight-relieving systems (e.g. Andago)
3 ☐Exoskeleton (e.g. Lokomat, ArmeoSpring)
4 ☐Exergames (e.g. Dividat Senso, Myro, DD System Elite Legpress, Wii, ReDance)
5 ☐Virtual reality (e.g. VR glasses, YouGrabber)
6 ☐Other: __________

Experience with the ExerCube system:
1 ☐Yes
2 ☐No

Training protocol

Handing over of the ExerG user handbook:

1 ☐Yes, date: ____________________
2 ☐No, why not :____________________

Form of the ExerG user handbook:
1 ☐Digital
2 ☐Printed

Training of the ExerG system:

Date: ____________________
Time: ____________________
Location: ____________________
Duration: ____________________
Initials of the person who carried out the training: ____________________

Figure S1: Flowchart of Decisions for Observing Secondary End Users' Use Scenarios.

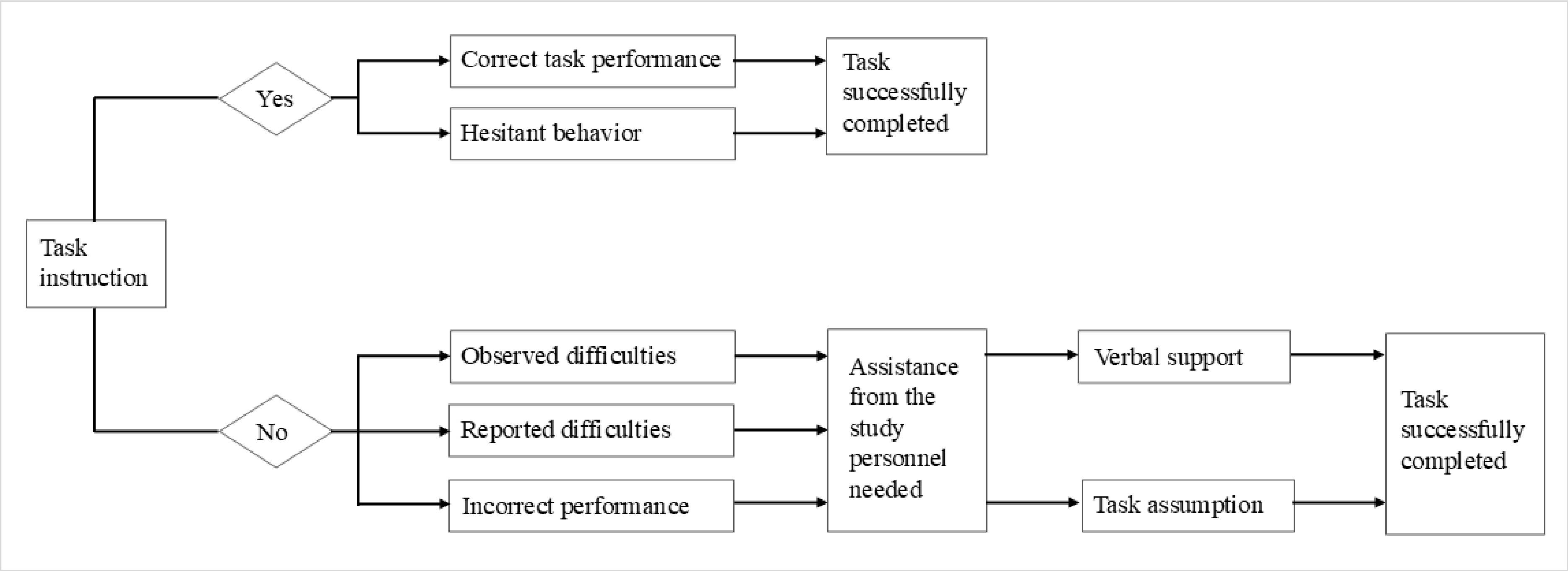

Observation Protocol

In the assessment, use checkboxes to indicate whether therapists perform the required tasks (yes, no), how they execute them (correctly, with hesitation, with difficulty, incorrectly), and whether they need assistance (verbal instruction or task completion by study personnel). Provide an empty column for comments and notes for each task. Record the time taken to complete each use scenario.

Preparing the hardware (Use Scenario 1)

No.	Task	Implementation	Support	Remarks (Difficulties or comments of the test persons)	Remarks (Difficulties or comments of the test persons)
1	Switching on the main power switch for the system	1 ☐Yes<br>2 ☐No	If yes,<br>1 ☐Correct<br>2 ☐Hesitant behavior<br>3 ☐Difficulties<br>4 ☐Incorrect<br>If no → support	1 ☐Yes<br>2 ☐No<br>If yes:<br>1 ☐Verbal assistance<br>2 ☐Task assumption by study staff	
2	Switching on the computer	1 ☐Yes<br>2 ☐No	If yes,<br>1 ☐Correct<br>2 ☐Hesitant behavior<br>3 ☐Difficulties<br>4 ☐Incorrect<br>If no → support	1 ☐Yes<br>2 ☐No<br>If yes:<br>1 ☐Verbal assistance<br>2 ☐Task assumption by study staff	
3	Switching on the touchscreen or checking whether it is already switched on	1 ☐Yes<br>2 ☐No	If yes,<br>1 ☐Correct<br>2 ☐Hesitant behavior<br>3 ☐Difficulties<br>4 ☐Incorrect<br>If no → support	1 ☐Yes<br>2 ☐No<br>If yes:<br>1 ☐Verbal assistance<br>2 ☐Task assumption by study staff	
4	Switching on the keypad or checking whether it is already switched on	1 ☐Yes<br>2 ☐No	If yes,<br>1 ☐Correct<br>2 ☐Hesitant behavior<br>3 ☐Difficulties<br>4 ☐Incorrect<br>If no → support	1 ☐Yes<br>2 ☐No<br>If yes:<br>1 ☐Verbal assistance<br>2 ☐Task assumption by study staff	
5	Switching on the three projectors using the remote control	1 ☐Yes<br>2 ☐No	If yes,<br>1 ☐Correct<br>2 ☐Hesitant behavior<br>3 ☐Difficulties<br>4 ☐Incorrect<br>If no → support	1 ☐Yes<br>2 ☐No<br>If yes:<br>1 ☐Verbal assistance<br>2 ☐Task assumption by study staff	

Continued on next page

No.	Task	Implementation	Support	Remarks (Difficulties or comments of the test persons)	Remarks (Difficulties or comments of the test persons)
6	Selecting the correct HDMI channels for the projectors using the remote control	1 ☐Yes<br>2 ☐No	If yes,<br>1 ☐Correct<br>2 ☐Hesitant behavior<br>3 ☐Difficulties<br>4 ☐Incorrect<br><br>If no → support	1 ☐Yes<br>2 ☐No<br><br>If yes:<br>1 ☐Verbal assistance<br>2 ☐Task assumption by study staff	
7	Entering the password for the computer	1 ☐Yes<br>2 ☐No	If yes,<br>1 ☐Correct<br>2 ☐Hesitant behavior<br>3 ☐Difficulties<br>4 ☐Incorrect<br><br>If no → support	1 ☐Yes<br>2 ☐No<br><br>If yes:<br>1 ☐Verbal assistance<br>2 ☐Task assumption by study staff	
8	Ensuring that the computer is connected to the Internet	1 ☐Yes<br>2 ☐No	If yes,<br>1 ☐Correct<br>2 ☐Hesitant behavior<br>3 ☐Difficulties<br>4 ☐Incorrect<br><br>If no → support	1 ☐Yes<br>2 ☐No<br><br>If yes:<br>1 ☐Verbal assistance<br>2 ☐Task assumption by study staff	

Total duration: __________

Preparing & Attaching the Safety System (Use Scenario 2)

In the assessment, use checkboxes to indicate whether therapists perform the required tasks (yes, no), how they execute them (correctly, with hesitation, with difficulty, incorrectly), and whether they need assistance (verbal instruction or task completion by study personnel). Provide an empty column for comments and notes for each task. Record the time taken to complete each use scenario.

No.	Task	Implementation	Support	Remarks (Difficulties or comments of the test persons)	Remarks (Difficulties or comments of the test persons)
1	Check that the rope is positioned correctly in all 9 deflection pulleys and is not damaged	1 ☐Yes<br>2 ☐No	If yes,<br>1 ☐Correct<br>2 ☐Hesitant behavior<br>3 ☐Difficulties<br>4 ☐Incorrect<br><br>If no → support	1 ☐Yes<br>2 ☐No<br><br>If yes:<br>1 ☐Verbal assistance<br>2 ☐Task assumption by study staff	
2	Performing the end stop test	1 ☐Yes<br>2 ☐No	If yes,<br>1 ☐Correct<br>2 ☐Hesitant behavior<br>3 ☐Difficulties<br>4 ☐Incorrect<br><br>If no → support	1 ☐Yes<br>2 ☐No<br><br>If yes:<br>1 ☐Verbal assistance<br>2 ☐Task assumption by study staff	
3	Correct donning of the Petzl Newton Fast harness system (in the correct size)	1 ☐Yes<br>2 ☐No	If yes,<br>1 ☐Correct<br>2 ☐Hesitant behavior<br>3 ☐Difficulties<br>4 ☐Incorrect<br><br>If no → support	1 ☐Yes<br>2 ☐No<br><br>If yes:<br>1 ☐Verbal assistance<br>2 ☐Task assumption by study staff	
4	Correct connection of the Petzl harness system	1 ☐Yes<br>2 ☐No	If yes,<br>1 ☐Correct<br>2 ☐Hesitant behavior<br>3 ☐Difficulties<br>4 ☐Incorrect<br><br>If no → support	1 ☐Yes<br>2 ☐No<br><br>If yes:<br>1 ☐Verbal assistance<br>2 ☐Task assumption by study staff	
5	Tightening the rope (if necessary)	1 ☐Yes<br>2 ☐No	If yes,<br>1 ☐Correct<br>2 ☐Hesitant behavior<br>3 ☐Difficulties<br>4 ☐Incorrect<br><br>If no → support	1 ☐Yes<br>2 ☐No<br><br>If yes:<br>1 ☐Verbal assistance<br>2 ☐Task assumption by study staff	

Total duration: __________

Simulating a Fall Scenario (Use Scenario 3)

In the assessment, use checkboxes to indicate whether therapists perform the required tasks (yes, no), how they execute them (correctly, with hesitation, with difficulty, incorrectly), and whether they need assistance (verbal instruction or task completion by study personnel). Provide an empty column for comments and notes for each task. Record the time taken to complete each use scenario.

No.	Task	Implementation	Support	Remarks (Difficulties or comments of the test persons)	Remarks (Difficulties or comments of the test persons)
6	Correct adjustment of the drop height using the crank handle	1 ☐Yes<br>2 ☐No	If yes,<br>1 ☐Correct<br>2 ☐Hesitant behavior<br>3 ☐Difficulties<br>4 ☐Incorrect<br><br>If no → support	1 ☐Yes<br>2 ☐No<br><br>If yes:<br>1 ☐Verbal assistance<br>2 ☐Task assumption by study staff	
7	Scenario 1: Patient falls and is caught by the safety system. To get back on your feet, you want to move the patient to a chair	1 ☐Yes<br>2 ☐No	If yes,<br>1 ☐Correct<br>2 ☐Hesitant behavior<br>3 ☐Difficulties<br>4 ☐Incorrect<br><br>If no → support	1 ☐Yes<br>2 ☐No<br><br>If yes:<br>1 ☐Verbal assistance<br>2 ☐Task assumption by study staff	
8	Scenario 2: Patient falls and is caught by the safety system. To get back on your feet, you want to lower the patient to the floor so that he/she can stand up on his/her own	1 ☐Yes<br>2 ☐No	If yes,<br>1 ☐Correct<br>2 ☐Hesitant behavior<br>3 ☐Difficulties<br>4 ☐Incorrect<br><br>If no → support	1 ☐Yes<br>2 ☐No<br><br>If yes:<br>1 ☐Verbal assistance<br>2 ☐Task assumption by study staff	

Total duration: __________

Initiating a Predefined Training Program (Use Scenario 4A)

In the assessment, use checkboxes to indicate whether therapists perform the required tasks (yes, no), how they execute them (correctly, with hesitation, with difficulty, incorrectly), and whether they need assistance (verbal instruction or task completion by study personnel). Provide an empty column for comments and notes for each task. Record the time taken to complete each use scenario.

Name of training sequence: Study training

No.	Task	Implementation	Support	**Remarks** (Difficulties or comments of the test persons)	**Remarks** (Difficulties or comments of the test persons)
1	Removing the trackers from the charging station	1 ☐Yes<br>2 ☐No	If yes,<br>1 ☐Correct<br>2 ☐Hesitant behavior<br>3 ☐Difficulties<br>4 ☐Incorrect<br><br>If no → support	1 ☐Yes<br>2 ☐No<br><br>If yes:<br>1 ☐Verbal assistance<br>2 ☐Task assumption by study staff	
2	Adequate attaching of the trackers to the (mock) patient's ankles and wrists	1 ☐Yes<br>2 ☐No	If yes,<br>1 ☐Correct<br>2 ☐Hesitant behavior<br>3 ☐Difficulties<br>4 ☐Incorrect<br><br>If no → support	1 ☐Yes<br>2 ☐No<br><br>If yes:<br>1 ☐Verbal assistance<br>2 ☐Task assumption by study staff	
3	Switching on the trackers (green light)	1 ☐Yes<br>2 ☐No	If yes,<br>1 ☐Correct<br>2 ☐Hesitant behavior<br>3 ☐Difficulties<br>4 ☐Incorrect<br><br>If no → support	1 ☐Yes<br>2 ☐No<br><br>If yes:<br>1 ☐Verbal assistance<br>2 ☐Task assumption by study staff	
4	Selecting the ExerG training program in the menu selection	1 ☐Yes<br>2 ☐No	If yes,<br>1 ☐Correct<br>2 ☐Hesitant behavior<br>3 ☐Difficulties<br>4 ☐Incorrect<br><br>If no → support	1 ☐Yes<br>2 ☐No<br><br>If yes:<br>1 ☐Verbal assistance<br>2 ☐Task assumption by study staff	
5	Selecting the predefined training sequence in the menu selection	1 ☐Yes<br>2 ☐No	If yes,<br>1 ☐Correct<br>2 ☐Hesitant behavior<br>3 ☐Difficulties<br>4 ☐Incorrect<br><br>If no → support	1 ☐Yes<br>2 ☐No<br><br>If yes:<br>1 ☐Verbal assistance<br>2 ☐Task assumption by study staff	

Continued on the next page

No.	Task	Implementation	Support	Remarks (Difficulties or comments of the test persons)	Remarks (Difficulties or comments of the test persons)
6	Starting the selected training program	1 ☐Yes<br>2 ☐No	If yes,<br>1 ☐Correct<br>2 ☐Hesitant behavior<br>3 ☐Difficulties<br>4 ☐Incorrect<br><br>If no → support	1 ☐Yes<br>2 ☐No<br><br>If yes:<br>1 ☐Verbal assistance<br>2 ☐Task assumption by study staff	
7	Correct guidance of the (mock) patient through the calibration	1 ☐Yes<br>2 ☐No	If yes,<br>1 ☐Correct<br>2 ☐Hesitant behavior<br>3 ☐Difficulties<br>4 ☐Incorrect<br><br>If no → support	1 ☐Yes<br>2 ☐No<br><br>If yes:<br>1 ☐Verbal assistance<br>2 ☐Task assumption by study staff	

Total duration: __________

Supporting a Predefined Training Program (Use Scenario 4B)

In the assessment, use checkboxes to indicate whether therapists perform the required tasks (yes, no), how they execute them (correctly, with hesitation, with difficulty, incorrectly), and whether they need assistance (verbal instruction or task completion by study personnel). Provide an empty column for comments and notes for each task. Record the time taken to complete each use scenario.

No.	Task	Implementation	Support	Remarks (Difficulties or comments of the test persons)	Remarks (Difficulties or comments of the test persons)
1	Accompanying the (mock) patient during the exergaming training	1 ☐Yes<br>2 ☐No	If yes,<br>1 ☐Correct<br>2 ☐Hesitant behavior<br>3 ☐Difficulties<br>4 ☐Incorrect<br><br>If no → support	1 ☐Yes<br>2 ☐No<br><br>If yes:<br>1 ☐Verbal assistance<br>2 ☐Task assumption by study staff	

Total duration: __________

Compilation of a Predefined Training Sequence (Use Scenario 5)

In the assessment, use checkboxes to indicate whether therapists perform the required tasks (yes, no), how they execute them (correctly, with hesitation, with difficulty, incorrectly), and whether they need assistance (verbal instruction or task completion by study personnel). Provide an empty column for comments and notes for each task. Record the time taken to complete each use scenario.

No.	Task	Implementation	Support	Remarks (Difficulties or comments of the test persons)	Remarks (Difficulties or comments of the test persons)
1	Selecting the ExerG training program in the launcher	1 ☐Yes<br>2 ☐No	If yes,<br>1 ☐Correct<br>2 ☐Hesitant behavior<br>3 ☐Difficulties<br>4 ☐Incorrect<br><br>If no → support	1 ☐Yes<br>2 ☐No<br><br>If yes:<br>1 ☐Verbal assistance<br>2 ☐Task assumption by study staff	
2	Opening a new training sequence	1 ☐Yes<br>2 ☐No	If yes,<br>1 ☐Correct<br>2 ☐Hesitant behavior<br>3 ☐Difficulties<br>4 ☐Incorrect<br><br>If no → support	1 ☐Yes<br>2 ☐No<br><br>If yes:<br>1 ☐Verbal assistance<br>2 ☐Task assumption by study staff	
3	Starting the editing mode	1 ☐Yes<br>2 ☐No	If yes,<br>1 ☐Correct<br>2 ☐Hesitant behavior<br>3 ☐Difficulties<br>4 ☐Incorrect<br><br>If no → support	1 ☐Yes<br>2 ☐No<br><br>If yes:<br>1 ☐Verbal assistance<br>2 ☐Task assumption by study staff	
4	Selecting appropriate exercises	1 ☐Yes<br>2 ☐No	If yes,<br>1 ☐Correct<br>2 ☐Hesitant behavior<br>3 ☐Difficulties<br>4 ☐Incorrect<br><br>If no → support	1 ☐Yes<br>2 ☐No<br><br>If yes:<br>1 ☐Verbal assistance<br>2 ☐Task assumption by study staff	
5	Naming the training sequence according to the specified patient ID	1 ☐Yes<br>2 ☐No	If yes,<br>1 ☐Correct<br>2 ☐Hesitant behavior<br>3 ☐Difficulties<br>4 ☐Incorrect<br><br>If no → support	1 ☐Yes<br>2 ☐No<br><br>If yes:<br>1 ☐Verbal assistance<br>2 ☐Task assumption by study staff	

Continued on the next page

No.	Task	Implementation	Support	Remarks (Difficulties or comments of the test persons)	Remarks (Difficulties or comments of the test persons)
6	Saving the training sequence	1 ☐Yes<br>2 ☐No	If yes,<br>1 ☐Correct<br>2 ☐Hesitant behavior<br>3 ☐Difficulties<br>4 ☐Incorrect<br><br>If no → support	1 ☐Yes<br>2 ☐No<br><br>If yes:<br>1 ☐Verbal assistance<br>2 ☐Task assumption by study staff	
7	Starting the newly created training sequence	1 ☐Yes<br>2 ☐No	If yes,<br>1 ☐Correct<br>2 ☐Hesitant behavior<br>3 ☐Difficulties<br>4 ☐Incorrect<br><br>If no → support	1 ☐Yes<br>2 ☐No<br><br>If yes:<br>1 ☐Verbal assistance<br>2 ☐Task assumption by study staff	

Total duration: __________

Exercise selection

Selection of exercises + level: | **Suitable exercise for the patient:**

- 1. ____________________ ☐1 Yes ☐2 No
-
- 2. ____________________ ☐1 Yes ☐2 No
-
- 3. ____________________ ☐1 Yes ☐2 No
-
- 4. ____________________ ☐1 Yes ☐2 No
-
- 5. ____________________ ☐1 Yes ☐2 No

Ending a Training Program (Use Scenario 6)

In the assessment, use checkboxes to indicate whether therapists perform the required tasks (yes, no), how they execute them (correctly, with hesitation, with difficulty, incorrectly), and whether they need assistance (verbal instruction or task completion by study personnel). Provide an empty column for comments and notes for each task. Record the time taken to complete each use scenario.

No.	Task	Implementation		Support	**Remarks** (Difficulties or comments of the test persons)
1	Ending the ExerG training program	1 ☐Yes<br>2 ☐No	If yes,<br>1 ☐Correct<br>2 ☐Hesitant behavior<br>3 ☐Difficulties<br>4 ☐Incorrect<br><br>If no → support	1 ☐Yes<br>2 ☐No<br><br>If yes:<br>1 ☐Verbal assistance<br>2 ☐Task assumption by study staff	
2	Exiting the user interface (launcher)	1 ☐Yes<br>2 ☐No	If yes,<br>1 ☐Correct<br>2 ☐Hesitant behavior<br>3 ☐Difficulties<br>4 ☐Incorrect<br><br>If no → support	1 ☐Yes<br>2 ☐No<br><br>If yes:<br>1 ☐Verbal assistance<br>2 ☐Task assumption by study staff	

Total duration: __________

Removing the Safety System (Use Scenario 7)

In the assessment, use checkboxes to indicate whether therapists perform the required tasks (yes, no), how they execute them (correctly, with hesitation, with difficulty, incorrectly), and whether they need assistance (verbal instruction or task completion by study personnel). Provide an empty column for comments and notes for each task. Record the time taken to complete each use scenario.

No.	Task	Implementation		Support	Remarks<br>(Difficulties or comments of the test persons)
1	Removing the wrist and ankle trackers	1 ☐Yes<br>2 ☐No	If yes,<br>1 ☐Correct<br>2 ☐Hesitant behavior<br>3 ☐Difficulties<br>4 ☐Incorrect<br>If no → support	1 ☐Yes<br>2 ☐No<br>If yes:<br>1 ☐Verbal assistance<br>2 ☐Task assumption by study staff	
2	Attaching the four trackers to the charging station	1 ☐Yes<br>2 ☐No	If yes,<br>1 ☐Correct<br>2 ☐Hesitant behavior<br>3 ☐Difficulties<br>4 ☐Incorrect<br>If no → support	1 ☐Yes<br>2 ☐No<br>If yes:<br>1 ☐Verbal assistance<br>2 ☐Task assumption by study staff	
3	Cranking down to release the tension on the rope	1 ☐Yes<br>2 ☐No	If yes,<br>1 ☐Correct<br>2 ☐Hesitant behavior<br>3 ☐Difficulties<br>4 ☐Incorrect<br>If no → support	1 ☐Yes<br>2 ☐No<br>If yes:<br>1 ☐Verbal assistance<br>2 ☐Task assumption by study staff	
4	Releasing the rope (connection to the Petzl harness system)	1 ☐Yes<br>2 ☐No	If yes,<br>1 ☐Correct<br>2 ☐Hesitant behavior<br>3 ☐Difficulties<br>4 ☐Incorrect<br>If no → support	1 ☐Yes<br>2 ☐No<br>If yes:<br>1 ☐Verbal assistance<br>2 ☐Task assumption by study staff	
5	Loosening and removing the Petzl Newton Fast harness system	1 ☐Yes<br>2 ☐No	If yes,<br>1 ☐Correct<br>2 ☐Hesitant behavior<br>3 ☐Difficulties<br>4 ☐Incorrect<br>If no → support	1 ☐Yes<br>2 ☐No<br>If yes:<br>1 ☐Verbal assistance<br>2 ☐Task assumption by study staff	

Total duration: __________

Terminating the Hardware (Use Scenario 8)

In the assessment, use checkboxes to indicate whether therapists perform the required tasks (yes, no), how they execute them (correctly, with hesitation, with difficulty, incorrectly), and whether they need assistance (verbal instruction or task completion by study personnel). Provide an empty column for comments and notes for each task. Record the time taken to complete each use scenario.

**No.**	**Task**	**Implementation**		**Support**	**Remarks** (Difficulties or comments of the test persons)
1	Switching off the three projectors using the remote control	1 ☐Yes<br>2 ☐No	If yes,<br>1 ☐Correct<br>2 ☐Hesitant behavior<br>3 ☐Difficulties<br>4 ☐Incorrect<br><br>If no → support	1 ☐Yes<br>2 ☐No<br><br>If yes:<br>1 ☐Verbal assistance<br>2 ☐Task assumption by study staff	
2	Shutting down the computer	1 ☐Yes<br>2 ☐No	If yes,<br>1 ☐Correct<br>2 ☐Hesitant behavior<br>3 ☐Difficulties<br>4 ☐Incorrect<br><br>If no → support	1 ☐Yes<br>2 ☐No<br><br>If yes:<br>1 ☐Verbal assistance<br>2 ☐Task assumption by study staff	
3	Switching off the main power switch of the system	1 ☐Yes<br>2 ☐No	If yes,<br>1 ☐Correct<br>2 ☐Hesitant behavior<br>3 ☐Difficulties<br>4 ☐Incorrect<br><br>If no → support	1 ☐Yes<br>2 ☐No<br><br>If yes:<br>1 ☐Verbal assistance<br>2 ☐Task assumption by study staff	

Total duration: __________

Procedure in specific situations with system problems or risks (knowledge tasks)

Knowledge Task 1: Interpretation of the intended purpose

No.	Situation	Correct procedure by the therapist described and shown (where feasible)?	Cause of incorrect response or incorrect procedure
1)	Please summarize the most important key points of the product's purpose in your own words	1 ☐Yes<br>2 ☐Yes, with the help of the manual<br>3 ☐No	1 ☐Information not available in the manual<br>2 ☐Information not clearly explained in the manual<br>3 ☐Other *(short description)*:

**Notes:**

Knowledge Task 2: Software System

No.	Situation	Correct procedure by the therapist described and shown (where feasible)?	Cause of incorrect response or incorrect procedure
1)	The volume of the video game is unpleasant.<br><br>How can you adjust the volume of the video game?	1 ☐Yes<br>2 ☐Yes, with the help of the manual<br>3 ☐No	1 ☐Information not available in the manual<br>2 ☐Information not clearly explained in the manual<br>3 ☐Other *(short description)*:
2)	The user interface does not open automatically after starting the computer.<br><br>What do you do if the user interface does not open immediately after you have started the computer?	1 ☐Yes<br>2 ☐Yes, with the help of the manual<br>3 ☐No	1 ☐Information not available in the manual<br>2 ☐Information not clearly explained in the manual<br>3 ☐Other *(short description)*:
3)	A person training has an incident, and the training should be stopped immediately.<br><br>How can you stop an ongoing training session immediately?	1 ☐Yes<br>2 ☐Yes, with the help of the manual<br>3 ☐No	1 ☐Information not available in the manual<br>2 ☐Information not clearly explained in the manual<br>3 ☐Other *(short description)*:
4)	A person exercising is too tired to perform the next exercise.<br><br>How can you skip an ongoing activity?	1 ☐Yes<br>2 ☐Yes, with the help of the manual<br>3 ☐No	1 ☐Information not available in the manual<br>2 ☐Information not clearly explained in the manual<br>3 ☐Other *(short description)*:

Continued on the next page

5)	The training program does not respond, and the training cannot be carried out.<br><br>How can you close and restart the training program?	1 ☐Yes<br>2 ☐Yes, with the help of the manual<br>3 ☐No	1 ☐Information not available in the manual<br>2 ☐Information not clearly explained in the manual<br>3 ☐Other *(short description)*:

**Notes:**

Knowledge Task 2: ExerCube Hardware

No.	Situation	Correct procedure by the therapist described and shown (where feasible)?	Cause of incorrect response or incorrect procedure
1)	One of the two HTC Vive cameras lights up red and training cannot be started.<br><br>What should you do, if one of the two cameras (in the corners) lights up "red"?	1 ☐Yes<br>2 ☐Yes, with the help of the manual<br>3 ☐No	1 ☐Information not available in the manual<br>2 ☐Information not clearly explained in the manual<br>3 ☐Other *(short description)*:
2)	One of the HTC Vive trackers lights up red, and the training cannot be started.<br><br>What should you do if one of the four trackers lights up red?	1 ☐Yes<br>2 ☐Yes, with the help of the manual<br>3 ☐No	1 ☐Information not available in the manual<br>2 ☐Information not clearly explained in the manual<br>3 ☐Other *(short description)*:
3)	The HTC Vive trackers are not recognized when the video game is started, and the calibration can therefore not be performed.<br><br>What do you need to check if the trackers are not recognized, when the video game is started, and therefore the calibration cannot be performed?	1 ☐Yes<br>2 ☐Yes, with the help of the manual<br>3 ☐No	1 ☐Information not available in the manual<br>2 ☐Information not clearly explained in the manual<br>3 ☐Other *(short description)*:
4)	The projection of the screens is reversed (e.g., the tablet screen appears on the front screen), or the surroundings are not projected correctly onto the screens (e.g., the borders of the screens do not correspond to the border of the projection, or the projection does not fill the entire screen).<br><br>What do you have to do if the projections are reversed or too small/too large?	1 ☐Yes<br>2 ☐Yes, with the help of the manual<br>3 ☐No	1 ☐Information not available in the manual<br>2 ☐Information not clearly explained in the manual<br>3 ☐Other *(short description)*:

Continued on the next page

Notes:

Knowledge Task 4: Backup system

No.	Situation	Correct procedure by the therapist described and shown (where feasible)?	Cause of incorrect response or incorrect procedure
1)	The rope is not correctly positioned in one of the deflection pulleys<br><br>How can you rectify this error?	1 ☐Yes<br>2 ☐Yes, with the help of the manual<br>3 ☐No	1 ☐Information not available in the manual<br>2 ☐Information not clearly explained in the manual<br>3 ☐Other *(short description)*:
2)	A strong resistance occurs during the end-stop test.<br><br>What steps do you need to take to check that the end stop is working correctly?	1 ☐Yes<br>2 ☐Yes, with the help of the manual<br>3 ☐No	1 ☐Information not available in the manual<br>2 ☐Information not clearly explained in the manual<br>3 ☐Other *(short description)*:

Notes:

Observation protocol interview

Difficulties with the system

- Follow-up questions in the event of hesitant behavior, difficulties and/or incorrect procedures
- What was the reason for the difficulties?
- Apart from the difficulties already mentioned, were there any other difficulties that were not observed?
- What would have helped to avoid the difficulty?

No.	Observed	Problem / task (description) / reason for difficulties
	1 ☐Yes<br>2 ☐No	
	1 ☐Yes<br>2 ☐No	
	1 ☐Yes<br>2 ☐No	

Continued on the next page

	1 ☐Yes<br>2 ☐No	
	1 ☐Yes<br>2 ☐No	
	1 ☐Yes<br>2 ☐No	
	1 ☐Yes<br>2 ☐No	

Continued on the next page

	1 ☐Yes<br>2 ☐No	
	1 ☐Yes<br>2 ☐No	
	1 ☐Yes<br>2 ☐No	
	1 ☐Yes<br>2 ☐No	

Semi-Structured Interview / Structured Questionnaire

Interaction with the System

Was the semi-structured interview conducted?

1 ☐Yes ☐2 No → Explanation: ____

Question	Answer
On a scale of 1-10, how satisfied were you with the use of the system?<br>(1= very dissatisfied, to<br>10 = very satisfied)	___________ *(score from 1-10)*
Would you like to use the system in your daily therapy routine?	1 ☐Yes, why *(brief explanation)*:<br><br>2 ☐No, why not *(brief explanation)*:
Do you think that the feedback in the video game was helpful/supportive for the person training?	1 ☐Yes<br>2 ☐In part, what else would be helpful in your opinion (*brief explanation*):

Continued on the next page

Do you think that the feedback in the video game was helpful/supportive for the person training?	3 ☐No, why not *(brief explanation)*:
Were you able to operate the system yourself with the information you received (training & user handbook)?	1 ☐Yes<br>2 ☐Partially<br>3 ☐No, why not *(brief explanation)*:
What physical functions or processes do you think the system trains?<br>*(List of possible functions and explanation)*	
In your opinion, do the processes that are trained with the system come close to everyday life processes?	1 ☐Yes<br>2 ☐Partially<br>3 ☐No, why not *(brief explanation)*:

Continued on the next page

Does the video game allow you to create a personalized training program?	1 ☐Yes<br>2 ☐Partially<br>3 ☐No, why not *(brief explanation)*:
Do you think the final screen of the video game provides a good summary of the trainee's performance	1 ☐Yes<br>2 ☐Partially<br>3 ☐No, why *(brief explanation)*:
Can you envision the trainee conducting the training independently after an initial session with a therapist, who is present to handle preliminary settings and safety system attachment if needed?	1 ☐Yes, why *(brief explanation)*:<br>2 ☐No, why *(brief explanation)*:

System Usability Scale

Was the SUS collected?
1 ☐Yes ☐2 No → Explanation: ____

	**For each of the following sentences, please indicate on a scale of 1 to 5 how much you agree or disagree with the statement.**	**Strongly disagree**	**Dis-agree**	**Neither agree nor disagree**	**Agree**	**Strongly agree**
1	I think that I would like to use this system frequently.	☐1	☐2	☐3	☐4	☐5
2	I found the system unnecessarily complex.	☐1	☐2	☐3	☐4	☐5
3	I thought the system was easy to use.	☐1	☐2	☐3	☐4	☐5
4	I think that I would need the support of a technical person to be able to use this system.	☐1	☐2	☐3	☐4	☐5
5	I found the various functions in this system were well integrated.	☐1	☐2	☐3	☐4	☐5
6	I thought there was too much inconsistency in this system.	☐1	☐2	☐3	☐4	☐5
7	I would imagine that most people would learn to use this system very quickly.	☐1	☐2	☐3	☐4	☐5
8	I found the system very cumbersome to use.	☐1	☐2	☐3	☐4	☐5
9	I felt very confident using the system.	☐1	☐2	☐3	☐4	☐5
10	I needed to learn a lot of things before I could get going with this system.	☐1	☐2	☐3	☐4	☐5

SUS score: __________

Device Deficiencies

No.	Type	Short description	Procedure	Led to SAE	Unex-pected
	1 ☐User interface<br>2 ☐Training settings<br>3 ☐Implementation of training<br>4 ☐Computer<br>5 HTC ☐Vive Tracker<br>6 HTC ☐Vive camera<br>7 ☐Beamer<br>8 ☐Computer<br>9 ☐Security system<br>10 ☐Other:___________		Could it be fixed?<br>1 ☐Yes<br>2 ☐Yes, with manual<br>3 ☐No<br>If no,<br>1 ☐Cancellation of the visit	1 ☐Yes<br>2 ☐No	1 ☐Yes<br>2 ☐No
	1 ☐User interface<br>2 ☐Training settings<br>3 ☐Implementation of training<br>4 ☐Computer<br>5 HTC ☐Vive Tracker<br>6 HTC ☐Vive camera<br>7 ☐Beamer<br>8 ☐Computer<br>9 ☐Security system<br>10 ☐Other:___________		Could it be fixed?<br>1 ☐Yes<br>2 ☐Yes, with manual<br>3 ☐No<br>If no,<br>1 ☐Cancellation of the visit	1 ☐Yes<br>2 ☐No	1 ☐Yes<br>2 ☐No
	1 ☐User interface<br>2 ☐Training settings<br>3 ☐Implementation of training<br>4 ☐Computer<br>5 HTC ☐Vive Tracker<br>6 HTC ☐Vive camera<br>7 ☐Beamer<br>8 ☐Computer<br>9 ☐Security system<br>10 ☐Other:___________		Could it be fixed?<br>1 ☐Yes<br>2 ☐Yes, with manual<br>3 ☐No<br>If no,<br>1 ☐Cancellation of the visit	1 ☐Yes<br>2 ☐No	1 ☐Yes<br>2 ☐No

Continued on the next page

	1 ☐User interface<br>2 ☐Training settings<br>3 ☐Implementation of training<br>4 ☐Computer<br>5 HTC ☐Vive Tracker<br>6 HTC ☐Vive camera<br>7 ☐Beamer<br>8 ☐Computer<br>9 ☐Security system<br>10 ☐Other:___________		Could it be fixed?<br>1 ☐Yes<br>2 ☐Yes, with manual<br>3 ☐No<br>If no,<br>1 ☐Cancellation of the visit	1 ☐Yes<br>2 ☐No	1 ☐Yes<br>2 ☐No
	1 ☐User interface<br>2 ☐Training settings<br>3 ☐Implementation of training<br>4 ☐Computer<br>5 HTC ☐Vive Tracker<br>6 HTC ☐Vive camera<br>7 ☐Beamer<br>8 ☐Computer<br>9 ☐Security system<br>10 ☐Other:___________		Could it be fixed?<br>1 ☐Yes<br>2 ☐Yes, with manual<br>3 ☐No<br>If no,<br>1 ☐Cancellation of the visit	1 ☐Yes<br>2 ☐No	1 ☐Yes<br>2 ☐No
	1 ☐User interface<br>2 ☐Training settings<br>3 ☐Implementation of training<br>4 ☐Computer<br>5 HTC ☐Vive Tracker<br>6 HTC ☐Vive camera<br>7 ☐Beamer<br>8 ☐Computer<br>9 ☐Security system<br>10 ☐Other:___________		Could it be fixed?<br>1 ☐Yes<br>2 ☐Yes, with manual<br>3 ☐No<br>If no,<br>1 ☐Cancellation of the visit	1 ☐Yes<br>2 ☐No	1 ☐Yes<br>2 ☐No
	1 ☐User interface<br>2 ☐Training settings<br>3 ☐Implementation of training<br>4 ☐Computer<br>5 HTC ☐Vive Tracker<br>6 HTC ☐Vive camera<br>7 ☐Beamer<br>8 ☐Computer<br>9 ☐Security system<br>10 ☐Other:___________		Could it be fixed?<br>1 ☐Yes<br>2 ☐Yes, with manual<br>3 ☐No<br>If no,<br>1 ☐Cancellation of the visit	1 ☐Yes<br>2 ☐No	1 ☐Yes<br>2 ☐No

Final Documentation

Date: __________ (MM/DD/YYYY)

Reason for ending the usability test:

1 ☐Scheduled completion of the test according to the test plan

2 ☐Withdrawal of consent

3 ☐Premature termination of the test by the manufacturer (Sphery)

4 ☐Other reason for early termination:

____

____

____
