# Supplementary material for: Exergame (ExerG)-Based Physical-Cognitive Training for Rehabilitation in Adults With Motor and Balance Impairments: Usability Study
Source: JMIR Serious Games. 2025 Feb 14;13:e66515. doi: 10.2196/66515 (PMC11844876; doi:10.2196/66515)
Supplement: Multimedia Appendix 5 [file games-v13-e66515-s005.pdf]

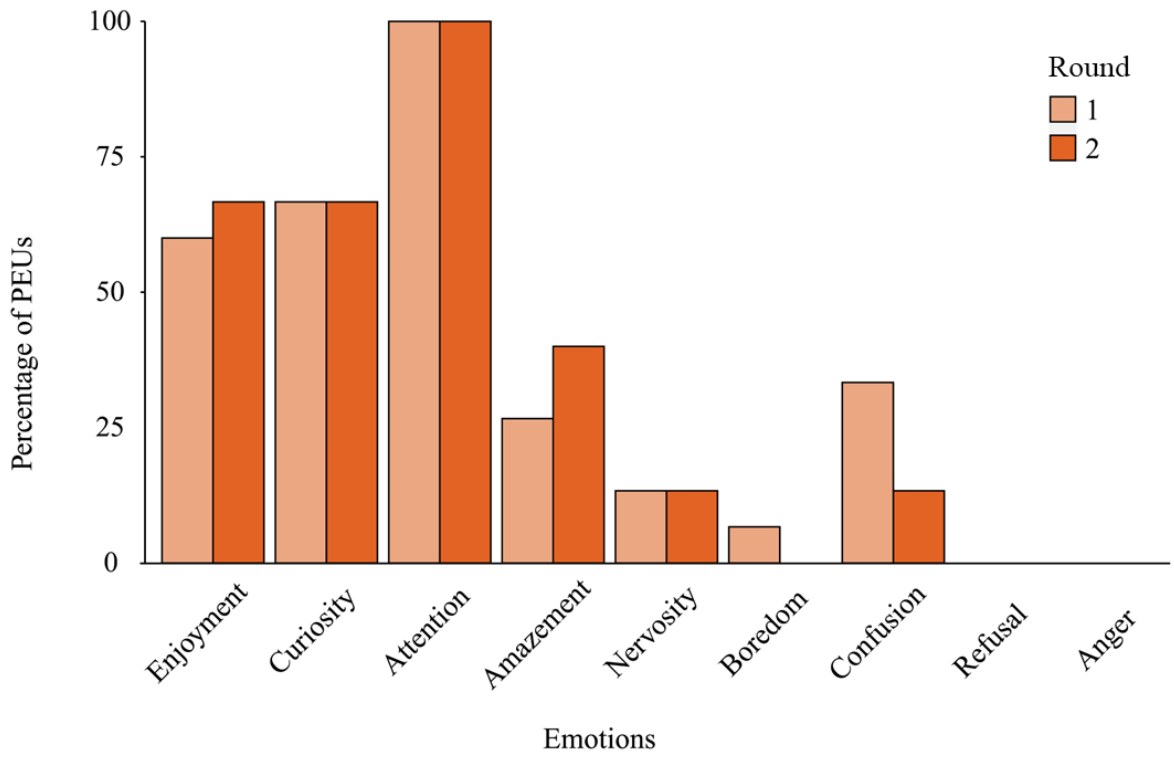

Figure S2. Patient-Expressed Emotions During Exercise Sessions.

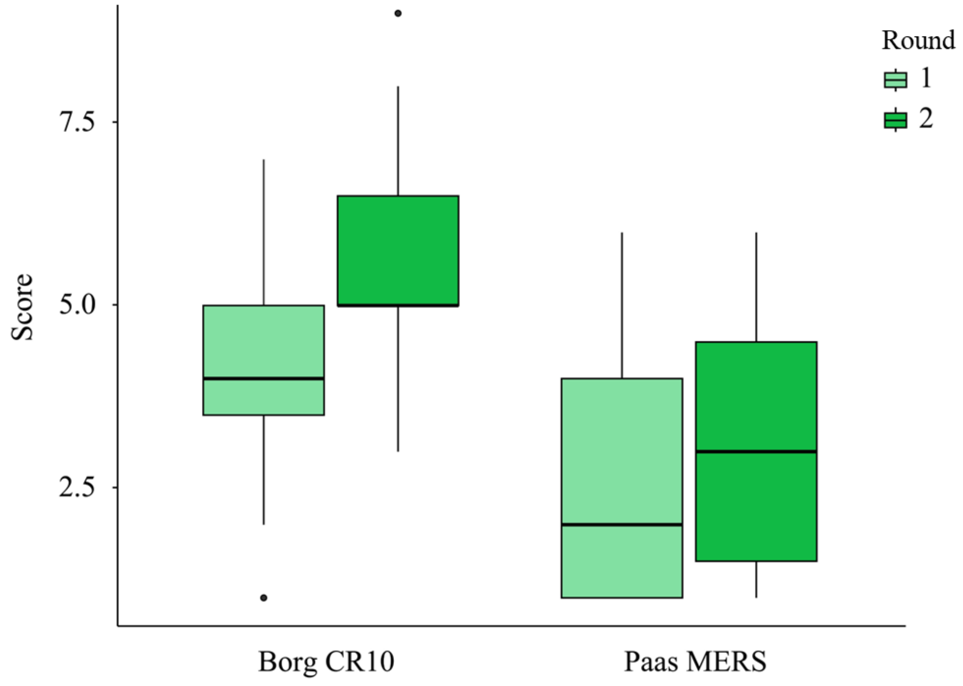

Figure S3. Physical and mental effort ratings.

Figure S3 presents a boxplot illustrating the reported physical and mental effort across patients. Each box represents the interquartile range (IQR), with the horizontal line inside the box indicating the median value. The whiskers extend to the minimum and maximum values within 1.5 times the IQR, while any points outside this range are marked as outliers.

Borg CR10: Borg Category Ratio 10 scale.
Paas MERS: Paas Mental Effort Rating Scale.

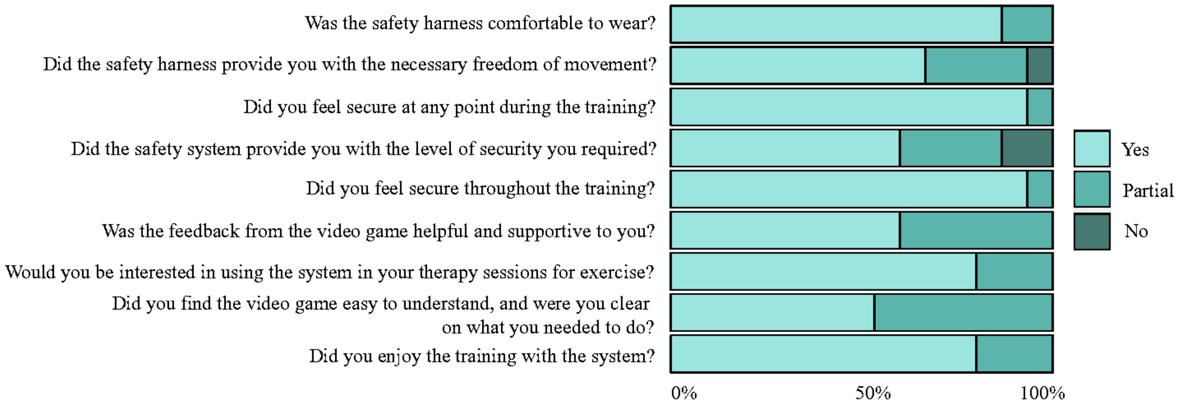

Figure S4. Feedback and Satisfaction of Patients with the ExerG from Structured Interviews.

Figure S4 presents a stacked bar chart illustrating the percentage of patients who responded with 'Yes,' 'Partial,' or 'No.' Each segment of the bars represents the proportion of each response category, allowing for a visual comparison of patient feedback.

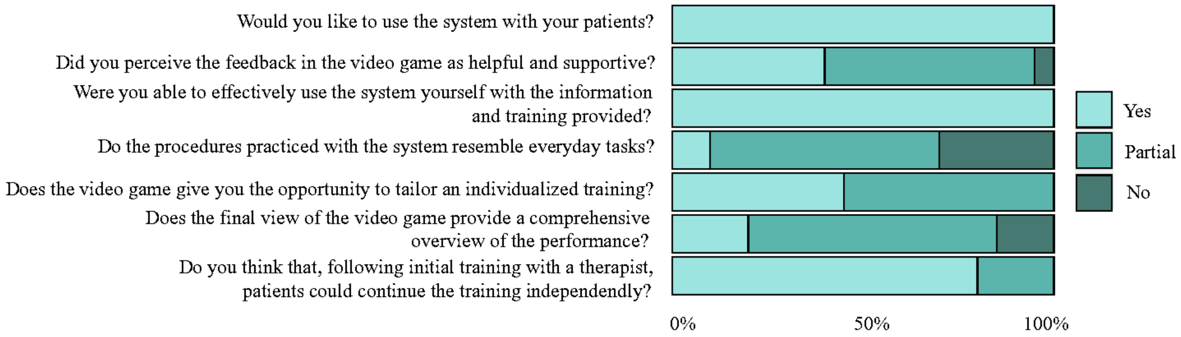

Figure S5. Results of structured interviews with therapists.

Figure S5 presents a stacked bar chart illustrating the percentage of therapists who responded with 'Yes,' 'Partial,' or 'No.' Each segment of the bars represents the proportion of each response category, allowing for a visual comparison of therapist feedback.
