# Supplementary material for: Exergame (ExerG)-Based Physical-Cognitive Training for Rehabilitation in Adults With Motor and Balance Impairments: Usability Study
Source: JMIR Serious Games. 2025 Feb 14;13:e66515. doi: 10.2196/66515 (PMC11844876; doi:10.2196/66515)
Supplement: Multimedia Appendix 6 [file games-v13-e66515-s006.pdf]

Table S1. Primary End User Theme Description

Theme	Description	Quote
Theme 1: Enjoyment and acceptance	Most patients reported that they enjoyed the training and expressed interest in using the device long-term, contingent upon specific improvements. These enhancements include better graphic design, an upgraded tracking system, an improved swivel arm, and higher-quality projectors. Moreover, providing a wider range of game activities and difficulty levels would enhance the overall experience.	'The training was fun, although the pixelation slightly detracted from the experience. Enhancing the game's design would make it even more enjoyable.' (PEU15)<br><br>'While the movement tracking had some delays, the training itself is very beneficial and supportive in my current situation.' (PEU01)
Theme 2: Live Interaction	In general, patients were able to read and comprehend the activity instructions and scoring feedback. They preferred shorter, more concise instructions with key task-related terms emphasized for clarity, as well as adequate time to read both the instructions and feedback.	'Activity instructions like 'tall grass walking' or 'rowing' would be even better if displayed for a longer period. Overall, making the instructions clearer and more precise would enhance the experience.' (PEU04)<br><br>'For some activities, receiving verbal instructions from the therapist made the instruction texts and activity descriptions much clearer.' (PEU06/PEU12)
Theme 3: Safety and Comfort	Patients consistently felt secure during the training sessions, primarily due to the comfortable safety harness. However, they recommended improvements to the safety system, as the abrupt movements of the swivel arm, due to its inertia, were unsettling and disrupted their balance, resulting in adjustments to their movement speed and execution. Some patients also experienced restrictions in navigating the entire game environment and found the rope pulley uncomfortable,	'I trained without the safety harness, but I can see how this safety system would be very helpful for others.' (PEU12)<br><br>'While using the safety system, I noticed it wasn't possible to reach the front corners of the cube. Addressing this would enhance the experience.' (PEU12)<br><br>'The swivel arm's pullback caused some swaying and slight balance issues. Refining this aspect would make the training smoother.' (PEU8)

	particularly during backward movements and jumping activities.	
