# Supplementary material for: Exergame (ExerG)-Based Physical-Cognitive Training for Rehabilitation in Adults With Motor and Balance Impairments: Usability Study
Source: JMIR Serious Games. 2025 Feb 14;13:e66515. doi: 10.2196/66515 (PMC11844876; doi:10.2196/66515)
Supplement: Multimedia Appendix 7 [file games-v13-e66515-s007.pdf]

Table S1. Secondary End User Theme Description

Themes	Description	Quote
Theme 1: Acceptance and motivation	Therapists noted that the video-game based training system has the potential to significantly boost patients' motivation for training, thanks to its appealing game design and surprising elements. The enjoyable and varied nature of the training contrasts positively with the routine of everyday therapy sessions.	'I believe this would significantly boost patients' motivation. Some patients are currently not very motivated and simply do not enjoy walking; some may not even want to be there. By incorporating a few natural elements and playful aspects, I think it could make the experience enjoyable for every patient, even those with cognitive impairments, enabling them to actively participate.' (SEU02)
Theme 2: Meaningful Activities	Therapists appreciated the game's activities for their benefits to overall physical and mental functions. They suggested that incorporating exercises mimicking daily living activities, such as walking on uneven terrain and handling real objects, could further enhance the training's significance and relevance.	'Walking on the spot is not an everyday movement.' (SEU02) 'From an occupational therapist's perspective, the movements of the upper extremities are not related to daily activities because approaching the objects, stretching or bending to reach it and grasping are not part of the training.' (SEU11)
Theme 3: Training feedback	Therapists found the audio-visual feedback and stars awarded at the end of each activity to be very helpful. Therapists recommended adding more motivating and specific feedback while reducing information overload. Simplifying the final overview by using easily classifiable values and total scores would enhance clarity and simplicity.	'Especially in the apple game, where you hear a sound when a new apple pops up, you know exactly where to look. This auditory cue serves as a training element and also provides a rewarding effect, enhancing the overall experience.' (SEU06) 'It would be helpful to have even more motivational phrases or reward effects during the game.' (SEU03)
Theme 4: Individualization	Therapists saw the system as a highly innovative training option suitable for diverse patient groups due to its integration of	'The ExerG trains balance, hand-eye coordination, motor functions, reaction, cognitive endurance, and attention.' (SEU05)

	cognitive and physical function training. They recommended expanding the exercise selection, enabling customization of training objectives, and tailoring the range of motion to individual needs for conditions like neglect or motor functions of a single upper extremity. Additionally, listing activities alphabetically with detailed descriptions would assist in selection and understanding.	'More or less customizable but choice of exercises should be greater and for stroke or hemiplegic patients it would be helpful to be able to define the range in which patients have to perform the activities (e.g. pick apples).' (SEU04)
Theme 5: Safety and Autonomy	The safety support materials were praised for providing a secure training environment, enabling challenging and limit-pushing training sessions. Further enhancements, such as improving the swivel arm construction to lessen the rebound effect, would optimize usability and safety. To support independent training, therapists suggested shorter, clearer, and more precisely formulated activity instructions that remain displayed until acknowledged. Independent training also depends on the patient's cognitive and physical condition.	'The risk of injuries is low and training beyond the limits is possible.' (SEU11)<br>'I think that independent training is possible as long as the patient is able to read and does not have cognitive impairment that reduces the ability to understand the instructions.' (SEU15) 'It depends on the physical and mental condition of the patients, their knowledge in robotic-assisted therapies and how safe they feel to train independently.' (SEU05)
